# Selective effect of cell membrane on synaptic neurotransmission

**DOI:** 10.1038/srep19345

**Published:** 2016-01-19

**Authors:** Pekka A. Postila, Ilpo Vattulainen, Tomasz Róg

**Affiliations:** 1 Department of Physics, Tampere University of Technology, P.O. Box 692, FI-33101 Tampere, Finland; 2 Department of Chemistry and Biochemistry, University of California San Diego, 92093-0340 San Diego, CA, USA; 3 MEMPHYS– Center for Biomembrane Physics, University of Southern Denmark, Odense, Denmark; 4 Department of Physics, University of Helsinki, P.O. Box 64, FI-00014, Helsinki, Finland

## Abstract

Atomistic molecular dynamics simulations were performed with 13 non-peptidic neurotransmitters (NTs) in three different membrane environments. The results provide compelling evidence that NTs are divided into membrane-binding and membrane-nonbinding molecules. NTs adhere to the postsynaptic membrane surface whenever the ligand-binding sites of their synaptic receptors are buried in the lipid bilayer. In contrast, NTs that have extracellular ligand-binding sites do not have a similar tendency to adhere to the membrane surface. This finding is a seemingly simple yet important addition to the paradigm of neurotransmission, essentially dividing it into membrane-independent and membrane-dependent mechanisms. Moreover, the simulations also indicate that the lipid composition especially in terms of charged lipids can affect the membrane partitioning of NTs. The revised paradigm, highlighting the importance of cell membrane and specific lipids for neurotransmission, should to be of interest to neuroscientists, drug industry and the general public alike.

Neurotransmission regulates a variety of senses and functions such as motivation, memory and muscle contraction. Neurotransmitters (NTs) play a key role in these functions since the signal transmission takes place through the binding of a NT with its specific receptor embedded in the postsynaptic membrane. Understanding the details of NT binding is important given that various diseases emerge if the signaling is impaired.

Currently, NTs are depicted in neurobiology textbooks to travel across the synaptic cleft over a distance of 20–40 nm and to bind to their receptors without interacting with the membrane itself^1^,^2^. However, several NT receptors (Figure S1, and Table S1 in the Supplementary Information (SI)) contain ligand-binding sites that are membrane-buried (Table 1), suggesting possible relevance of the cell membrane in the NT binding.

Until a few decades ago, membranes were considered as passive participants in cellular processes. Recently, however, lipids and membranes have been shown to be in a central position in numerous cellular functions such as signaling and protein sorting^3^. The conformation and dynamics as well as the function of various membrane-embedded proteins has been shown to be dependent on the lipid composition surrounding membrane proteins^4^,^5^,^6^. Specific lipids can modulate the stability and function of membrane-embedded proteins by diffusing into specific binding sites on their surfaces^7^,^8^. For example, cardiolipins have been suggested to facilitate proton transfers during cytochrome *bc*_1_ complex operation^9^,^10^,^11^,^12^,^13^ by binding to locations close to the enzyme’s active sites in the mitochondrial membrane^9^,^10^,^11^.

The effect of lipids and cell membranes is not limited to proteins and peptides but the dynamics of ligands and other small molecules is affected, too. For instance, antipsychotic drugs have been shown via numerous biophysical methods to interact strongly with lipid membranes^12^. Membrane permeability is a feature known to influence bioavailability of drugs, and major experimental and theoretical effort has been invested to estimate ligand permeability across membranes^13^,^14^. Furthermore, analgesic drugs such as propofol and even NTs have been suggested to produce anesthetic effects by affecting membrane properties such as bilayer thickness^15^,^16^. The biological effect is, therefore, not necessarily a result of direct ligand-receptor interaction, but the proteins’ functions can be altered via ligand-induced changes in its membrane environment^17^. To this end, a growing number of both experimental and computational studies exploring ligand-membrane interactions are emerging^18^,^19^,^20^.

A substantial body of work indicates that some ligands first bind to the membrane surface and then diffuse laterally into the membrane-buried binding sites of their receptors^21^. In these cases, the direct 3D diffusion of the ligands from the water phase to the receptor’s binding site is transformed into 3D-2D diffusion, which speeds up the binding process by several orders of magnitude^21^. Despite this, the potentially significant effect that the lipid composition exerts on ligand-receptor entry kinetics in the synapse has been largely ignored in previous studies. For example with ionotropic glutamate receptors (Table S1), valuable pharmacological data can be acquired by concentrating only on their extracellular ligand-binding domains^22^,^23^,^24^,^25^,^26^,^27^. However, when studying for instance acetylcholine entry into the M_3_ muscarinic acetylcholine receptor, the lipid composition is a factor that should not be overlooked^28^. Although the positively charged NT is not expected to partition to the cell membrane based on its hydrophilic profile, it is yet able to bind effectively into the membrane-buried binding sites of muscarinic receptors as well as into the extracellular binding sites of acetylcholinesterase and nicotinic acetylcholine receptors^28^.

Several experimental and computational studies have indicated that the lipid composition in membranes has a role in NT dynamics. The lipid composition has been shown by molecular dynamics (MD) simulations and calorimetry^29^,^30^ to affect the NT-membrane dynamics with dopamine. The same result has been shown also for glutamate, acetylcholine, γ-aminobutyric acid (GABA) and glycine using MD simulations, calorimetry, and dialysis equilibrium experiments^31^,^32^. With serotonin, melatonin, and peptidic NT encephalin, attachment to neutral membranes has been also documented by means of neutron scattering^33^, MD simulations^34^,^35^,^36^, and nuclear magnetic resonance measurements^36^. In general, the presence of anionic lipids and the zwitterionic state of NTs enhances membrane attachment. Further, determining the role of membrane environment for NT binding in the synapses is clearly relevant given that lipids occupy roughly half of the cellular membrane surface area, while the other half is taken up by various proteins^37^.

In this work we performed atomistic MD simulations for three lipid bilayer models with 13 non-peptidic NTs and the dopamine precursor L-dopa. The pre-equilibrated lipid membrane models (64 lipids/leaflet; see SI) reproduce experimentally determined local bilayer properties such as the order parameter, membrane thickness and average area per lipid (e.g, ref. 38,); accordingly, the models are suitable for studying NT-membrane interactions that take place at the water-membrane interface.

The first “extracellular” membrane model represents the extracellular leaflet of a synaptic membrane, being composed of dioleoylphosphatidylcholine, sphingomyelin, and cholesterol (DOPC/SM/CHOL)^37^. The second “intracellular” model represents a more polar intracellular leaflet and contains dilinoleicphosphatidylcholine, dilinoleicphosphatidylethanolamine, and dilinoleicphosphatidylserine (DLPC/DLPE/DLPS)^37^. Since the exact contents of postsynaptic membranes are not known, the lipid compositions of these two models follow the lipid contents of typical animal cell membranes. Finally, the third “control” model, containing only DLPC lipids, was used as a less polar control membrane. Altogether, 42 NT-membrane systems were simulated for 200–400 ns each; furthermore, the membrane binding energetics were determined for four individual NTs using the umbrella sampling method.

Based on the MD simulation results the current view of neurotransmission, which does not account for the role of lipids^1^,^2^, was revised and a holistic theory highlighting the selective effect of cell membrane on synaptic neurotransmission was formulated. In short, the simulations provide strong evidence that neurotransmission follows either the membrane-independent or the membrane-dependent mechanism, and the one chosen by nature depends on the position of the receptor’s ligand-binding site.

## Results

**Figure 1:**
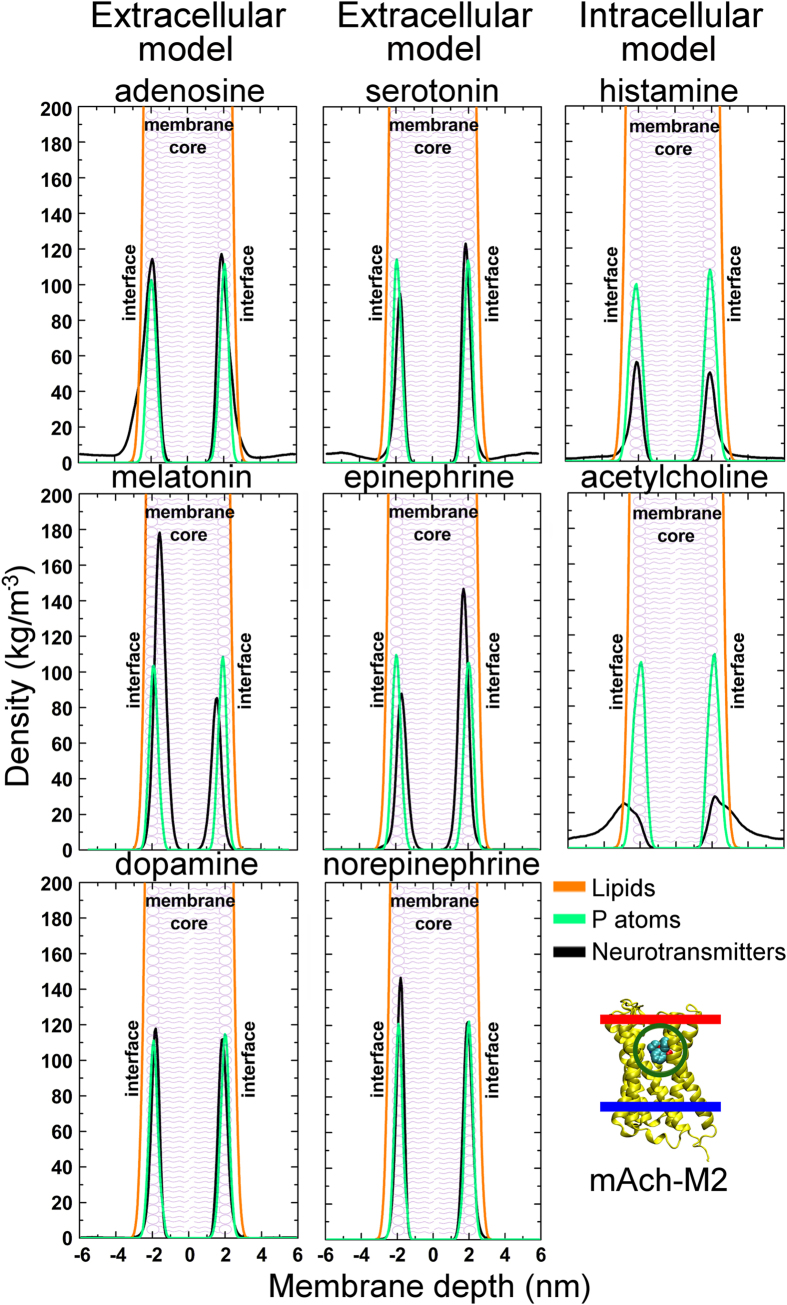
Density profiles for neurotransmitters with membrane buried binding sites. The density curves indicate that adenosine, epinephrine, melatonin, and dopamine attached reversibly to the extracellular leaflet surface, and the positively charged acetylcholine and histamine attached to the polar intracellular leaflet. In the bottom right corner, extracellular (red) and intracellular (blue) membrane sides and the ligand-binding site (green circle) are indicated with lines for the structure of the muscarinic acetylcholine receptor (Table S1).

**Figure 2:**
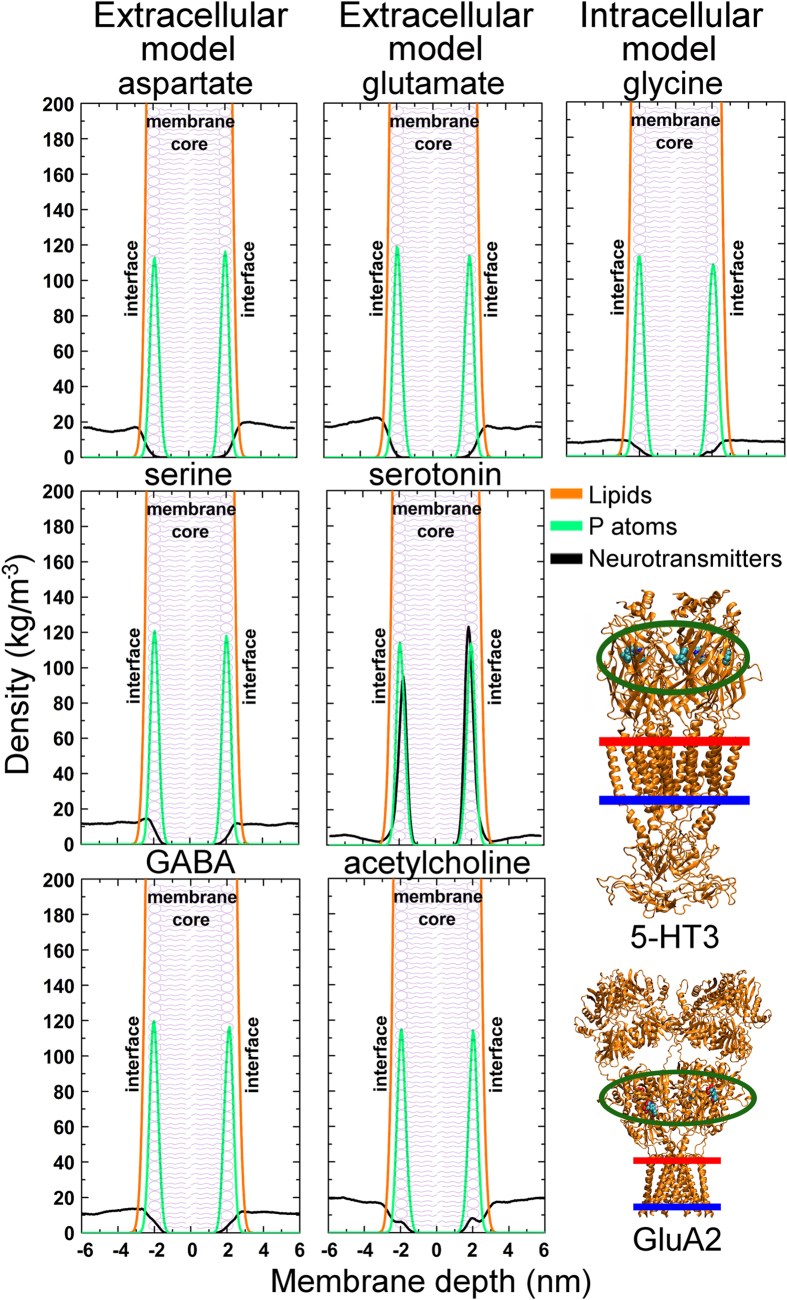
Density profiles for neurotransmitters with extracellular binding sites. The density curves indicate that aspartate, glutamate, serine, glycine, GABA, and acetylcholine do not attach to the extracellular leaflet. For serotonin, there exist receptors with membrane-buried binding sites and extracellular binding sites (Table 1), but the NT attached to all membranes. Also shown (on the right, bottom) are the extracellular binding sites of the GluA2 glutamate receptor and the 5HT-3 serotonin receptor (Table S1).

As expected, the NTs did not permeate the lipid bilayers; instead they displayed varying degrees of reversible membrane attachment. Catecholamines such as dopamine and norepinephrine, as well as serotonin, melatonin, and adenosine, all attached strongly to the lipid bilayers in the NT-membrane simulations (Fig. 1; Table 1). The hydrophobic ring systems were found to be essential for the favorable alignment on the membrane surface. In comparison, small NTs containing charged moieties, such as GABA or glutamate (Fig. 2; Table 1), did not attach to the membranes. Hydrogen bonding was prominent when NTs attached to the models of the intracellular and extracellular leaflets. With the less polar control membrane, hydrophobic/steric attraction was required for close contact. The specific NT-membrane interactions are discussed thoroughly in the SI (Figures S3–S11 and Tables S2–S4).

**Figure 3:**
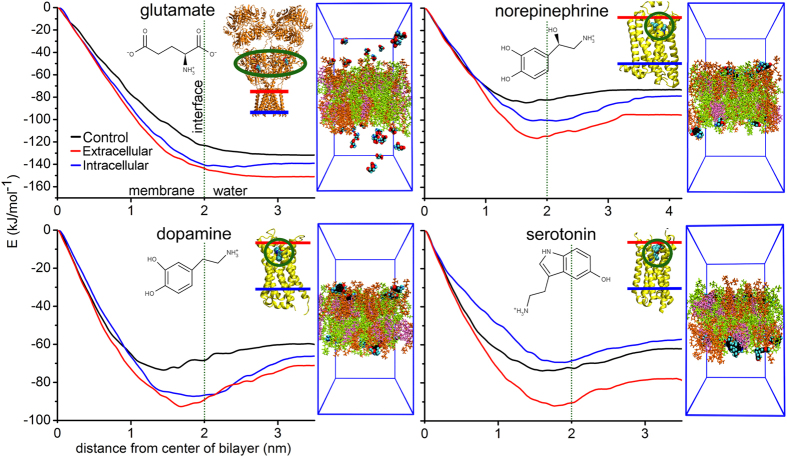
Profiles of free energy obtained from umbrella sampling calculations. The profiles indicate that norepinephrine, dopamine and serotonin adhere to the membrane surfaces, while glutamate does not. Both the 2D structures of NTs and the 3D receptor structures are shown in the figures. For comparison, the NT-membrane reversible attachment is observed in the end of the extracellular leaflet simulations, too.

**Figure 4:**
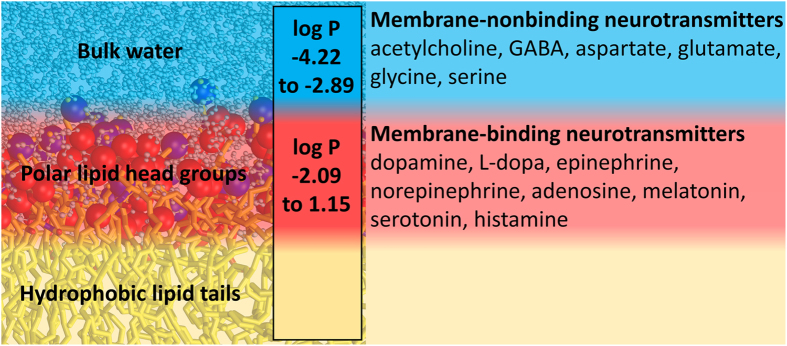
Lipophilicity classifies neurotransmitters into membrane-nonbinding and membrane-binding categories.

The simulations indicate that NTs can be divided into membrane-binding (Fig. 1) and membrane-nonbinding groups (Fig. 2). The MD simulations (Table 1), free energy computations (Fig. 3; Table S5), and log P (the octanol/water partition coefficient) values of the NTs (Fig. 4; Table S3) support this view. Although octanol clearly cannot depict all of the physical properties and the diversity of an actual lipid bilayer, the log P values corroborate the simulation results (Table S3). What is more, this categorization of NTs corresponds to the positioning of the ligand-binding sites of their receptors in relation to the postsynaptic membrane (Tables 1 and S1). As a rule of thumb, if a NT attached to a membrane, then its ligand-binding site is membrane-buried. In contrast, whenever the ligand-binding site is extracellular, then the NT did not attach to the membrane (Table 1).

**Figure 5:**
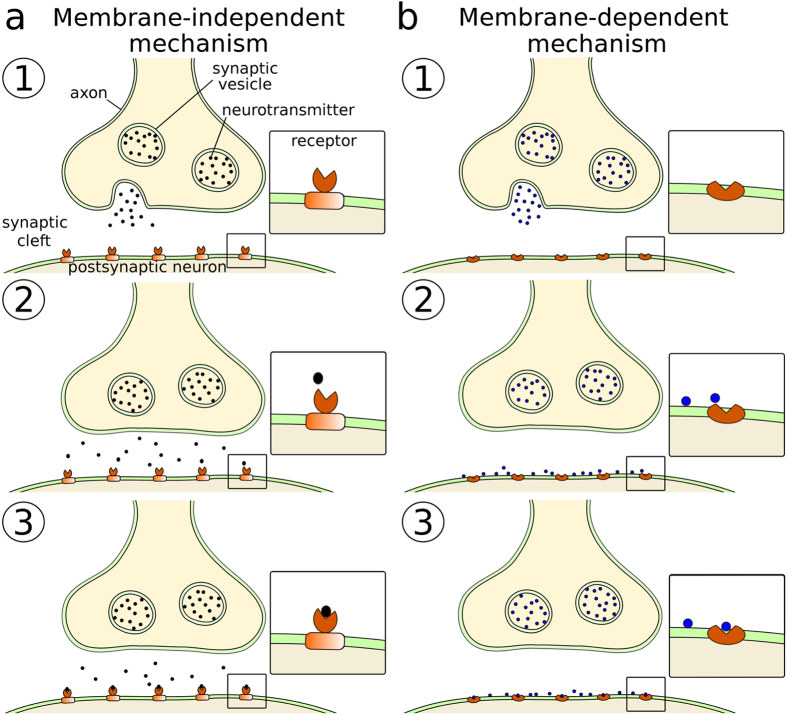
Neurotransmission models. (**a**) Classical membrane-independent mechanism: (1) neurotransmitter release; (2) random diffusion; and (3) binding into the extracellular ligand-binding sites of the receptors. (**b**) Membrane-dependent mechanism: (1) neurotransmitter release; (2) aggregation to the membrane; and (3) lateral diffusion followed by binding into the membrane-buried ligand-binding sites.

Based on the divergent behavior observed in the simulations, neurotransmission can be split into two different mechanisms (Fig. 5). The classical membrane-independent mechanism (Fig. 5a), shown in neurobiology textbooks, dictates that NTs, whose receptors’ ligand-binding sites are extracellular, travel across the synaptic cleft without adhering to the membrane. Thus, these NTs enter their binding site directly from the bulk water. The membrane-dependent mechanism (Fig. 5b) proposed in this work dictates that NTs, whose binding sites are located in the transmembrane part of the receptor, reversibly attach first to the membrane and then diffuse along the membrane surface to the membrane-buried binding site.

## Discussion

Although the lipid types residing in synapses are known, the exact lipid compositions of specific synapses or, more importantly, their extracellular and intracellular leaflets are yet to be characterized^39^,^40^,^41^,^42^. Keeping this in mind, let us consider the perspectives emerging from the simulation data for specific lipid types’ role in the context of NT binding into proteins embedded at the postsynaptic membrane.

The ligand-binding sites of G protein-coupled receptors are typically membrane-buried (Table 1) and their representative NTs follow the membrane-dependent mechanism (Fig. 5b). Adenosine, epinephrine, norepinephrine, dopamine, serotonin, and melatonin were found to attach reversibly predominantly to the extracellular and intracellular leaflets in the MD simulations (Table 1), stressing the preference for lipid binding. The positively charged histamines attached preferentially to the DLPS-containing intracellular leaflet instead of the extracellular membrane surface (Table 1; Figures S8,S9), which suggests that negatively charged lipids could be more abundant in synapses housing histamine receptors. For serotonin, there exist several 5-HT receptors with membrane-buried binding sites but also 5-HT3 ion channel receptors with extracellular ligand-binding sites (Figs 1,2, S6 and S7; Table 1). As serotonin was found to adhere to all three membrane models, assuring sufficient amount of NT binding into the extracellular binding sites of 5-HT3 receptors might require for example relatively high serotonin levels.

The ligand-gated ion channels such as ionotropic glutamate receptors have extracellular ligand-binding sites. Accordingly, their representative NTs follow the membrane-independent mechanism (Fig. 5a). GABA, glycine, serine, glutamate, and aspartate prefer the bulk water to the extracellular leaflet (Table 1). Interestingly, in the cases of serine and acetylcholine, the polar intracellular leaflet was preferred to the bulk water (Figures S8,S9). The fact that acetylcholine attaches to the intracellular surface, and not to the extracellular leaflet, provides an explanation for how muscarinic acetylcholine receptors can have membrane-buried ligand-binding sites while nicotinic acetylcholine receptors have extracellular binding sites (Table 1). Namely, those synapses or membrane regions populated by muscarinic receptors could yet again have more negatively charged lipids attracting the positively charged acetylcholine molecules than those populated by nicotinic receptors. Altogether, the results suggest that specific lipids, and charged types in particular, may modulate the association of NTs to their host membranes, and hence the binding to their receptors.

The above described results suggest that specific lipid types and the lipid composition in general could have an important role in synaptic neurotransmission. While the concentrations of negatively charged glycolipids are usually small in typical animal cells, their levels are known to be higher in neurons^39^,^40^. Thus, these anionic lipids at the extracellular leaflet of the postsynaptic membrane could facilitate the membrane attachment of positively charged histamine or acetylcholine molecules. While the effect of glycolipids was not addressed in this work, the simulations indicate that charged lipids (here DLPS) do affect the partitioning of NTs (Table 1).

Tightly regulated lipid composition differences between synapses could ensure that acetylcholine can bind into both membrane-buried and extracellular ligand-binding sites (Table 1; Figures S8,S9). In fact, the lipid composition may also be an important factor in neurological diseases and/or their diagnostics. Post mortem studies have indicated changes in the lipid composition with schizophrenia, Parkinson’s disease, and Alzheimer’s disease patients^43^,^44^,^45^,^46^. The membrane reversible attachment of a NT could be either enhanced or weakened, if the membrane became less or more charged due to changes in lipid composition. Although the absolute NT levels would not necessarily be affected by these changes, an imbalance in NT-membrane interactions could contribute to disease states such as depression.

Synaptic receptors are not the only membrane-embedded proteins that interact with NTs at the postsynaptic membrane. The timely removal of NTs from the synaptic cleft is vital and, for example, if glutamate transport is obstructed, neurotoxic effects follow^47^. In this regard, the NT-membrane simulations shed new light also on NT transport across the postsynaptic membrane following neurotransmission.

It may seem contradictory, but all NTs eventually bind into the membrane-buried ligand-binding sites of their transporters (Figure S12a,b). The Na^+^ gradient across the membrane powers the Na^+^/NT co-transport. In addition, the positive ion(s) at the ligand-binding sites form ionic bonds with NTs that have carboxylate groups (shown for leucine in Figure S12b)^48^. With monoamines such as serotonin, which partition readily to the membrane (Table 1), an aspartate side chain acts as an intrinsic ligand which neutralizes the positive charge^48^. In the glutamate transporter the Na^+^ binding takes place prior to the NT entry^49^. Thus, the positively charged ions inside the membrane-buried ligand-binding sites of NT transporters could act as sinks that attract negatively charged NTs such as glutamate and aspartate.

This sink hypothesis is not valid for neutral ligands, but all non-peptidic NTs were somewhat attracted to the polar intracellular membrane in the simulations, serine even preferred it over the bulk water (Figures S10,S11; Table 1). Conceivably, the NT transporters could be surrounded by high concentrations of anionic lipids such as glycolipids. Indeed, lipid composition differences have been suggested to affect, for example, glutamate uptake^50^. Notably, the removal of acetylcholine from the synaptic cleft is different as the positively charged NT binds into the extracellular ligand-binding site of acetylcholinesterase (Figure S12c) and the reaction products are transported separately into the cytoplasm.

In a broader context, the simulations suggest a strong interplay between lipids, membrane proteins, and NTs, and coevolution at the postsynaptic membrane. The NTs not only have to pass the on/off test of the membrane and match the properties of their receptors’ active sites in the synapse, but the membrane effect likely applies to the intracellular NT dynamics as well. However, it is important to recognize that drugs or natural compounds such as toxins do not necessarily follow the same logic as the NTs regarding the membrane partitioning. For example, potent acetylcholinesterase inhibitors can be very lipophilic despite the fact that the enzyme’s active site is extracellular (Table S6). This discrepancy highlights the extraordinary importance of fast and coordinated NT kinetics for neurotransmission. Despite this, avoiding ligand-membrane mismatches during drug development should reduce off-target effects and increase efficacy with a multitude of target proteins.

In conclusion, the atomistic MD simulations suggest that non-peptidic NTs are divided into membrane-binding and membrane-nonbinding categories that in turn correspond to the positioning of their receptor’s ligand-binding sites in relation to the postsynaptic membrane (Table 1). Hence, neurotransmission follows either the membrane-dependent or the membrane-independent mechanism (Fig. 5). Despite the apparent simplicity of this finding, it represents a fundamentally different outlook on neurotransmission with far-reaching ramifications. The selective effect of the membrane ensures a more coordinated response to NT release than random diffusion could accomplish alone. By controlling the lipid composition, and paying attention to the extracellular leaflet in particular in different synapses in the nervous system, NTs such as acetylcholine could bind efficiently into membrane proteins with both extracellular and membrane-buried ligand-binding sites. Moreover, anionic lipids promote the membrane partitioning of all studied non-peptidic NTs in the simulations (Table 1) and, thus, the presence of negatively charged lipids in the synapse could be a requirement for efficient removal of NTs from the synaptic cleft.

## Methods

The atomistic molecular dynamics (MD) simulations were performed with GROMACS 4.5^51^ using the OPLS-AA force field^38^,^52^,^53^. The MD simulation protocol was presented in a previous study^38^. Free energy curves were calculated using umbrella sampling^54^ together with the Weighted Histogram Analysis Method^55^. VMD1.9.1 was used to draw the 3D structures of receptors^56^. Altogether, 42 NT-membrane systems were simulated, each for 200–300 ns (total simulation time being ~12 μs). The computational methods are explained in detail in SI.

## Additional Information

**How to cite this article**: Postila, P. A. *et al.* Selective effect of cell membrane on synaptic neurotransmission. *Sci. Rep.* **6**, 19345; doi: 10.1038/srep19345 (2016).

## Supplementary Material

Supplementary Information

## Figures and Tables

**Table 1:**
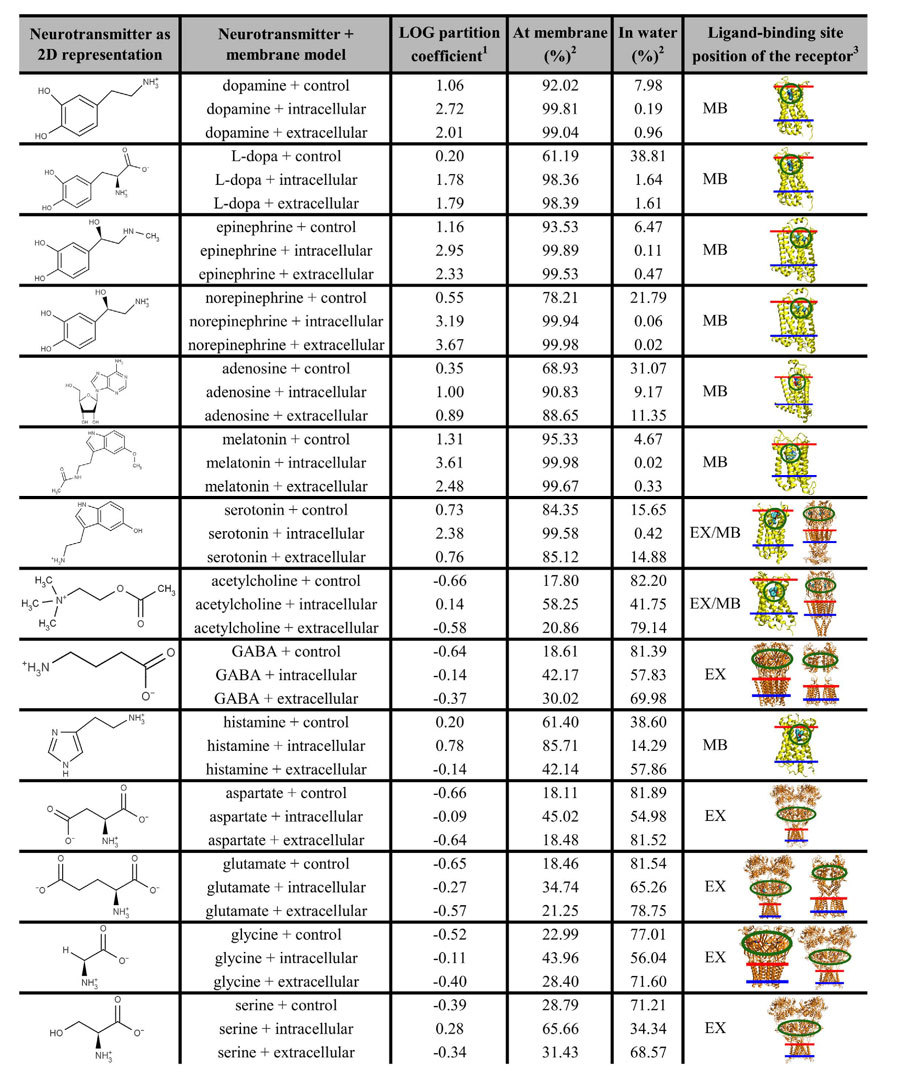
The neurotransmitter-membrane association.

^1^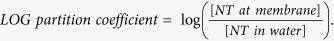 ^2^NT was considered bound to the membrane, if it resided within +/−1 nm from the head group nitrogen atom’s average position along the normal to the bilayer surface of either bilayer leaflet. ^3^The receptors have extracellular (EX) and/or membrane-buried (MB) binding sites (Table S1). The binding sites are circled (green). The extracellular (red) and intracellular (blue) surfaces are indicated with lines.
