# Supplementary material for: Selective effect of cell membrane on synaptic neurotransmission
Source: Sci Rep. 2016 Jan 19;6:19345. doi: 10.1038/srep19345 (PMC4725992; doi:10.1038/srep19345)
Supplement: Supplementary Information [file srep19345-s1.pdf]

Supporting Information For

Selective effect of cell membrane on synaptic neurotransmission

Pekka A. Postila[1,2], Ilpo Vattulainen[1,3,4], Tomasz Róg[1]*

[1]Department of Physics, Tampere University of Technology, P.O. Box 692, FI-33101 Tampere, Finland

[2]Department of Chemistry and Biochemistry, University of California San Diego, 92093-0340 San Diego, CA, USA

[3]MEMPHYS – Center for Biomembrane Physics, University of Southern Denmark, Odense, Denmark

4 Department of Physics, University of Helsinki, P.O. Box 64, FI-00014, Helsinki, Finland

*Corresponding author: tomasz.rog@tut.fi

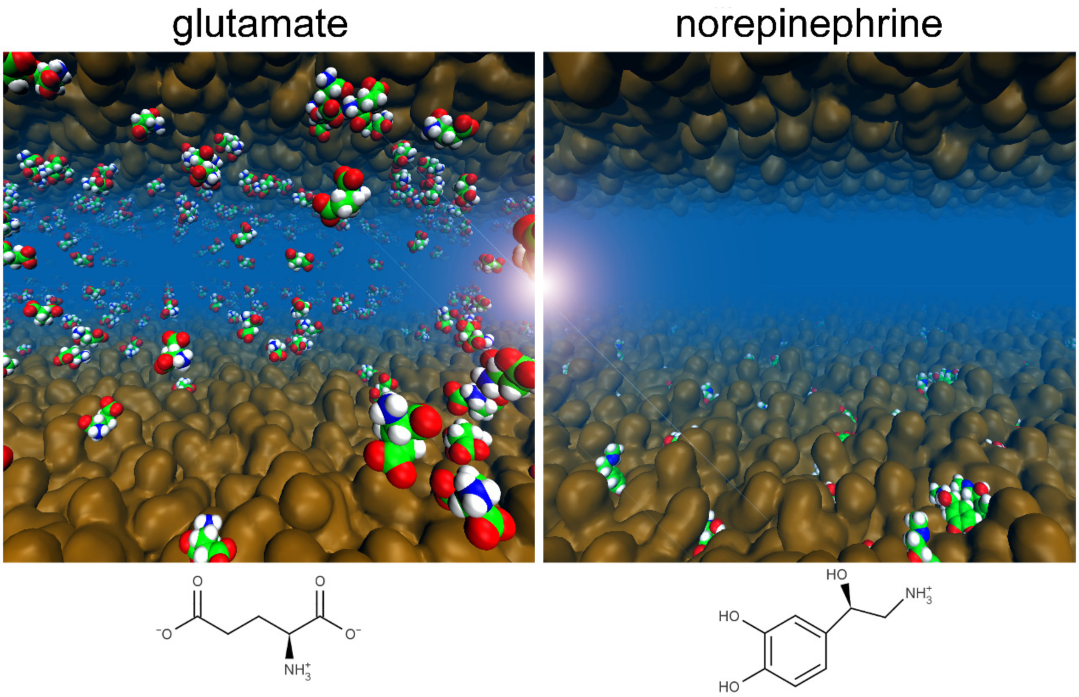

Table of Contents

Text 1. General Outline of the Supporting Information.

Text 2. Expanded Methods.

Text 3. Catecholamines Form Tight Association with the Membrane.

Text 4. Predictive Power of the Octanol/Water Partition Coefficient.

Text 5. Serotonin and Melatonin Attach Strongly to the Membrane.

Text 6. Histamine-Membrane Association at an Intermediate Level.

Text 7. Amino Acids Do Not Bind to the Membrane.

Text 8. Free Energy of Profiles.

Text 9. Supplementary References.

Figure S1. 2D structures of non-peptidic neurotransmitters.

Figure S2. Schematic representations of membrane lipids.

Figure S3. The amount of membrane hydrogen bonding per neurotransmitter.

Figure S4. Neurotransmitter-membrane densities with group I molecules.

Figure S5. Neurotransmitter-membrane associations with group I molecules.

Figure S6. Neurotransmitter-membrane densities with group II molecules.

Figure S7. Neurotransmitter-membrane association with group II molecules.

Figure S8. Neurotransmitter-membrane densities with group III molecules.

Figure S9. Neurotransmitter-membrane association with group III molecules.

Figure S10. Neurotransmitter-membrane densities with group IV molecules.

Figure S11. Neurotransmitter-membrane association with group IV molecules.

Figure S12. Profiles of free energy obtained from umbrella sampling calculations.

Figure S13. Role of membrane when turning down the neurotransmitter-mediated signal.

Table S1. Synaptic neurotransmitter receptor subtypes.

Table S2. Membrane hydrogen bonding per neurotransmitter and area per lipid values.

Table S3. Predicted and experimental water/octanol partition coefficients.

Table S4. The partition coefficient ranges of neurotransmitters follow ligand-binding site positioning.

Table S5. Free energy difference between bulk water and the water-membrane interface for neurotransmitters.

Table S6. Predicted water/octanol partition coefficients for active DUD compounds.

Text 1. General Outline of the Supporting Information.
For clarity, the simulated non-peptidic neurotransmitters (NTs) were divided into groups I-IV (Figure S1). The details, including hydrogen-bonding (H-bonding) analysis, density profiles and the simulation end states of all NT-membrane molecular dynamics (MD) simulations are presented according to this same division. The sequence of the supporting figures and tables follows the order they are cited in the article.

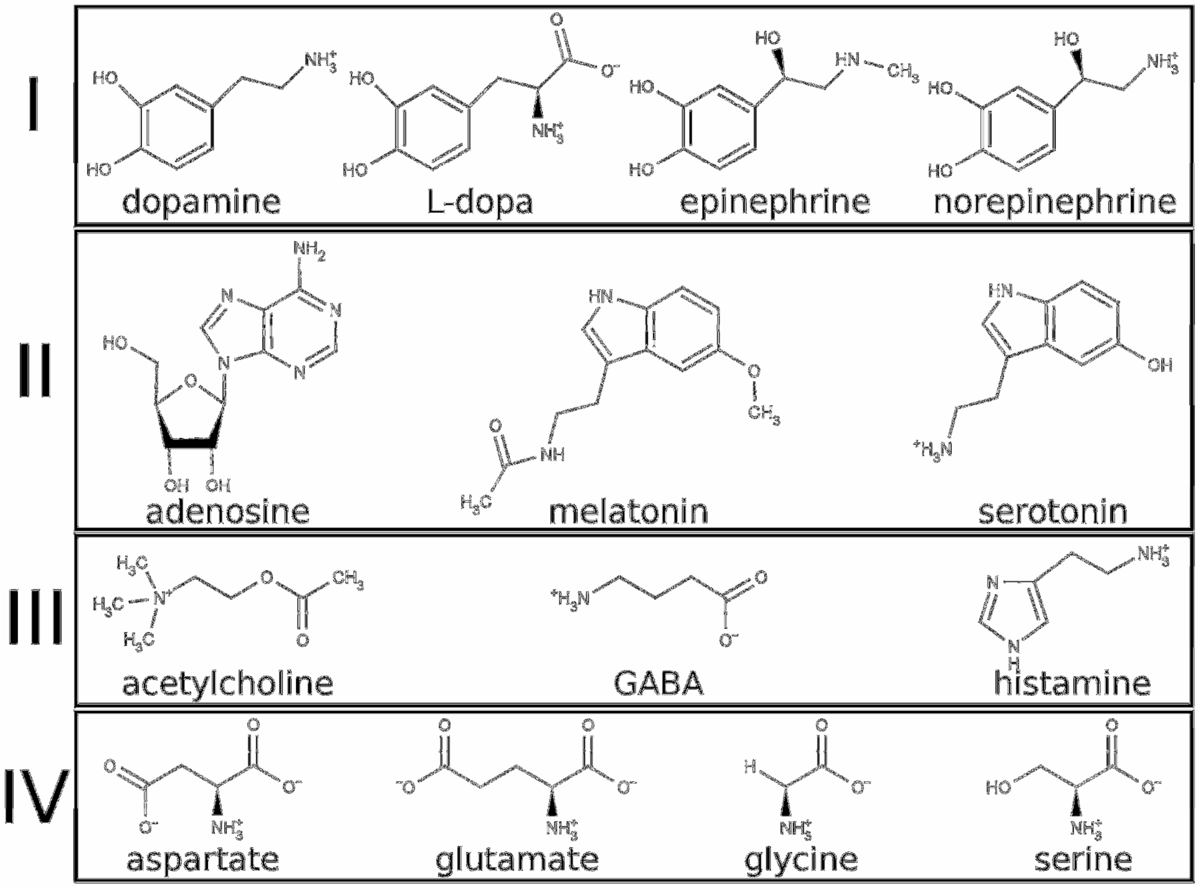

Figure S1. 2D structures of non-peptidic neurotransmitters. The simulated NTs are divided into four groups (I-IV) based on their structural features: I) dopamine, its precursor L-dopa, epinephrine, and norepinephrine; II) adenosine, melatonin, and serotonin; III) acetylcholine, γ-aminobutyric acid (GABA), and histamine; IV) aspartate, glutamate, glycine, and serine.

Text 2. Extended Methods.
System build-up. 13 non-peptidic NTs, including acetylcholine (Ach), adenosine, aspartate, dopamine, epinephrine (or adrenaline), γ-aminobutyric acid (GABA), glutamate, glycine, histamine, melatonin, norepinephrine (or noradrenaline), serine, and serotonin (or 5-hydroxytryptamine) and dopamine precursor L-dopa were simulated in this study. The NTs were drawn in 3D using AVOGADRO1.03 (1) and their protonation was set to match pH 7.4 (Figure S1). The NTs were geometry optimized and electrostatic potentials were calculated with GAUSSIAN09A.01 (Gaussian Inc., Wallingford, CT, USA) at the HF/6-31G* level with the PCM water model. The charges were fitted using RBRA set up

in RED-vIII (2). When possible, the NT topology information was derived directly from the equivalent amino acids of the OPLSAA force field (3; 4).

Three phospholipid bilayer membrane models (128 lipids in each system) were used in this study. 1) The DOPC/SM/CHOL model was composed of dioleoylphosphatidylcholine (DOPC; n=48; Figure S2), sphingomyelin (SM; n=48; Figure S2) and cholesterol (CHOL; n=32; Figure S2). This composition corresponds to the extracellular leaflet of the animal cell membrane and it also mimics the extracellular leaflet composition of the postsynaptic membrane. 2) The DLPC/DLPE/DLPS model included dilinoleicphosphatidyl choline (DLPC, di-18:2-DLPC; n=44; Figure S2), dilinoleic-phosphatidylethanolamine (DLPE, di-18:2-PE; n=60; Figure S2) and dilinoleicphosphatidylserine (DLPS, di-18:2PS; n=24; Figure S2). It represents the intracellular leaflet of the postsynaptic cell membrane or presynaptic vesicle (5). 3) The DLPC model, which functioned as a control membrane, contained only dilinoleicphosphatidylcholine (DLPC, di-18:2-DLPC; n=128; Figure S2).

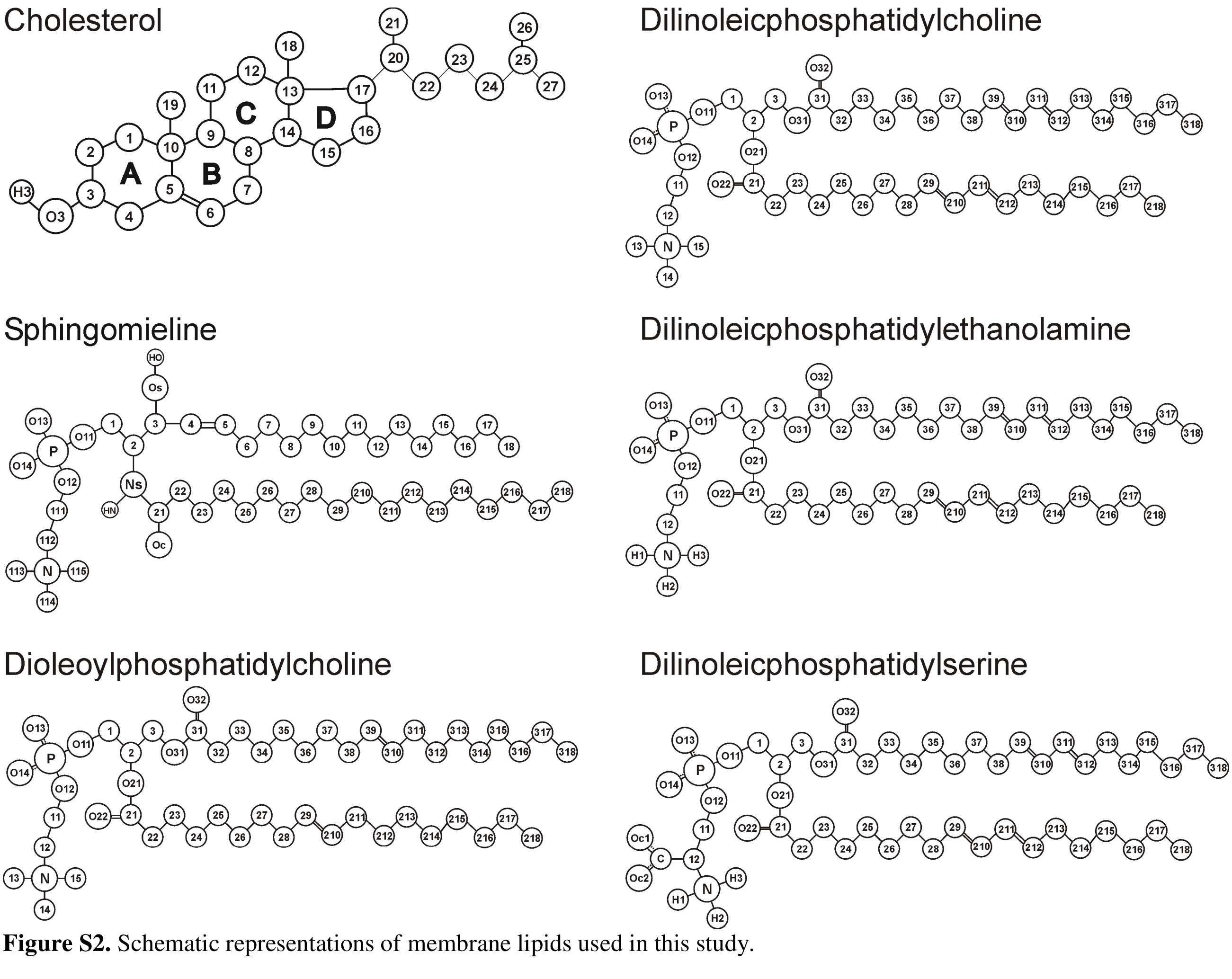

Figure S2. Schematic representations of membrane lipids used in this study.

In total, 20 NT molecules (Figure S1) were randomly positioned in the water phase using BODIL0.8 (6) on both sides of the bilayer. This corresponds to ~100 mM NT concentration. Each system was solvated with OPLS compatible TIP3P water molecules (7) and the net charge was neutralized using either Na^+ or Cl^- ions. The system dimensions were 6 nm x 6 nm x 12 nm in XYZ directions. In total, 42 NT-membrane systems (Table S2) were simulated classically in this study.

Molecular dynamics simulations. The atomistic molecular dynamics (MD) simulations were performed using GROMACS 4.5 (8) and the OPLS-AA force field (3; 4) as described in our previous study (9). Periodic boundary conditions were applied in all dimensions with the usual minimum image convention. LINCS algorithm was used to preserve the length of hydrogen atom covalent bonds (10). A time step of 2 fs was used in the simulations; however, melatonin and serotonin were simulated with a 1 fs time step to avoid bond angle problems. A constant pressure of 1 bar and temperature of 310 K were maintained using the Parrinello-Rahman and Nosé-Hoover methods, respectively (11; 12). The solute and solvent temperatures were controlled separately. A cut off at 1 nm was used for the Lennard-Jones interactions while the particle mesh Ewald method (13) was used for the electrostatic interactions. Each system was energy minimized using the steepest descent algorithm before running the 200-400 ns production MD simulations. The system was regarded equilibrated once the H-bonding between the NTs and lipids leveled off. Only the equilibrated parts of the trajectories were used for further analysis.

Umbrella sampling simulations. Umbrella sampling was performed with glutamate, dopamine, serotonin, and norepinephrine with all three membrane models to determine the location of their energy minima in relation to the membrane, i.e., whether they prefer the membrane surface or the bulk water phase. First, the NT in question was pulled into the center of the lipid bilayer from the water phase using steered molecular dynamics simulation. An umbrella pulling force constant of 1000 kJ mol^{-1} nm^{-1} and a pull rate of 0.01 nm/ps were used to move the NT in the Z direction using the center of mass of the lipid bilayer as a reference (origin at Figure 2c). Secondly, snapshot windows with 0.1 nm interval were extracted from the trajectory to cover the entry of the NT from bulk water into the membrane center. Third, the NT was constrained at the Z direction using harmonic umbrella potential (1000 kJ mol^{-1} nm^{-1}) and the system was MD simulated. Finally, fourth, the weighted histogram analysis method (WHAM) was used to acquire the potential of mean force or binding free energy for the NT (14). The first 5 ns of the trajectories collected for the umbrella windows were discarded to assure that only equilibrated parts of the trajectories were used in the analysis. The elongation of the trajectories was stopped once extra steps did not produce noticeable changes to the plotted free energy curves. In general, 44 umbrella windows were simulated for 25-80 ns each. Sufficient window overlap was determined by inspecting WHAM histograms, and statistical errors were estimated with bootstrap analysis.

Figure preparation. GROMACS package tools were used to calculate NT-membrane H-bonding, average area per lipid values and density profiles from the simulation trajectories. The 3D structures of supplementary tables and figures were generated using VMD1.9.1 (15). The NT 2D structures shown in in the supplementary figures and tables were drawn using MARVINSKETCH 5.4.1 (ChemAxon,

Inc., Budapest, Hungary). The 3D structures were acquired from the Protein Data Bank (PDB) (16) for following receptors: adenosine receptor A2A (PDB: 2YDO) (17), dopamine receptor D3 (human; PDB: 3PBL) (18), epinephrine or β2-adrenergic receptor (PDB: 3D4S) (19), histamine H1 receptor (PDB: 3RZE) (20), serotonin or 5-hydroxytryptamine 5-HT1B receptor (human; PDB: 4IAQ) (21), ionotropic glutamate GluA2 receptor (*Rattus norvegicus*; PDB: 3KG2) (22), bacterial leucine transporter (*Aquafex aeolicus*; PDB: 2A65) (23), acetylcholinesterase (*Homo sapiens*; PDB: 4BDT) (24), monoamine oxidase B (*Homo sapiens*; PDB: 2XFN) (25), muscarinic acetylcholine receptor M2 (PDB: 3UON) (26), and nicotinic acetylcholine receptor (*Torpedo marmorata*; PDB: 2BG9) (27). Nicotine molecules were superimposed for the acetylcholine receptor from the X-ray crystal structure of Ach binding protein (*Lymnaea stagnalis*; PDB: 1UW6) (28). The boundaries of the receptor's transmembrane parts were indicated based on the information presented in the Orientations of Proteins in Membrane (OPM) database (29). Melatonin receptor MT2 homology model was acquired from a study by Pala et al. (30) 5-HT3A homology model (Uniprot: P46099), which is based on the X-ray structure of nicotinic acetylcholine receptor (PDB: 2BG9), was acquired from the SWISSMODEL Repository (31; 32). $GABA_A$-$\alpha_1\beta_2\gamma_2$ receptor homology model, which is based on X-ray structures of the glutamate-gated chloride channel (PDB: 3RIF) and the ligand-gated ion channel from *Erwinia chrysanthemi* (ELIC; PDB: 2VL0) (33), was acquired from a study by Bergmann et al. (34). The glycine receptor GlyR-α1/β homology model shown was also acquired from the SWISSMODEL Repository. Both subunits α1 (Uniprot: P23415) and β (Uniprot: P48167) were build using an X-ray structure of the pentameric glutamate-gated chloride channel (PDB: 3RHW) (35). The ionotropic glutamate receptor GluN1/2A homology model was build using Homodge in BODIL0.8 (6). Both GluN1 (Uniprot: Q05586) and GluN2A (Uniprot: Q12879) subunits were aligned and tetrameric model was build using the X-ray structure of ionotropic glutamate receptor GluA2 (PDB: 3KG2). The ionotropic glutamate receptor GluK2 homology model (Uniprot: Q13002) was built similarly using Homodge and the GluA2 tetramer X-ray structure (PDB: 3KG2). The extracellular ligand-binding domains of metabotropic glutamate receptor mGluR1 (*Rattus norvegicus*; PDB: 1EWK) (36) and $GABA_{B1}$ receptor (*Homo sapiens*; PDB: 4F11) (37) were positioned above the membrane (*Homo sapiens*; PDB: 4OR2) (38) in roughly the same orientation as in GluA2 receptor (PDB: 3KG2) without homology modelling.

Text 3. Catecholamines Form Tight Association with the Membrane.
Group I molecules or catecholamines, which include dopamine, its precursor L-dopa, epinephrine, and norepinephrine, contain benzene-1,2 diol structures as a scaffold. Accordingly, the differences in their level of membrane association must arise from their different C4-moieties (Figure S1). The ligand-binding sites of the catecholamine NTs are membrane-buried as is typical for G protein-coupled receptors (Table S1). By looking at the amount of direct H-bonding between these NTs and the membrane models it is evident that they all have a strong tendency to adhere to the membrane surfaces (Figure S3; Table S2). Regardless of the simulated catecholamine the H-bonding was the weakest with the least polar control membrane (DLPC; Figure S2). In contrast, the H-bonding was at the highest level with the extracellular model leaflet (DOPC/SM/CHOL; Figure S2) for all catecholamines except for L-dopa that bound slightly better to the intracellular leaflet (DLPC/DLPE/DLPS; Figure S2). Norepinephrine-membrane H-bonding was stronger than that of the other compounds with all three

membranes, while dopamine produced the second highest score. L-dopa produced the third highest amount of H-bonding. However, epinephrine was able to interact substantially stronger with the control membrane than L-dopa. Density profiles also indicate that catecholamines had a close association with all membrane models (Figure S3 and Table 1). The four ligands adhered better to the extracellular leaflet than to the intracellular leaflet (black lines in Figure S3; Table 1). With the control membrane catecholamines settled relatively deep into the bilayer. This membrane-buried arrangement was needed to accomplish optimal hydrophobic interactions with the less polar DLPC lipids. Equally deep entry into the DLPC membrane could only be seen with melatonin, serotonin and histamine (see below). This buried arrangement was not seen with L-dopa (Figure S3), because its negatively charged carboxylate group restricted its alignment. The strong catecholamine-membrane association was also seen when inspecting the last simulation frames with both the extracellular and intracellular leaflets (Figure S4).

Table S1. Synaptic neurotransmitter receptor subtypes.

Synaptic receptor name	Receptor subtypes	Neurotransmitters	Neurotransmitter as 2D representation[1]	Mechanism of action[2]	Ligand-binding site position[3]	
adenosine receptor	A_1, $A_{2A\text{-}B}$, A_3	adenosine	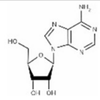	GPCR	MB	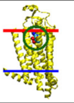
adrenergic receptor	ADR-$\alpha_{1A,B,D}$, $\alpha_{2A\text{-}C}$, $\beta_{1\text{-}3}$	epinephrine, norepinephrine	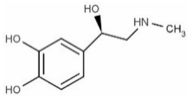	GPCR	MB	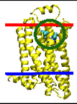
dopamine receptor	$D_{1\text{-}5}$	dopamine	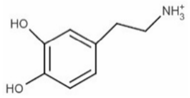	GPCR	MB	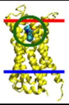
histamine receptor	H1-4	histamine	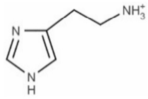	GPCR	MB	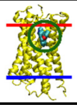
melatonin receptor	MT1-3	melatonin	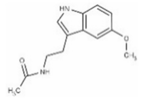	GPCR	MB	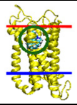
serotonin receptor	5-$HT_{1A\text{-}B}$, 5-$HT_{1D\text{-}F}$, 5-$HT_{2A\text{-}C}$, 5-HT_4, 5-$HT_{5A\text{-}B}$, 5-$HT_{6\text{-}7}$	serotonin	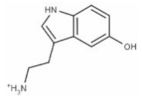	GPCR	MB	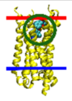
muscarinic acetylcholine receptor	mAch-M1-5	acetylcholine	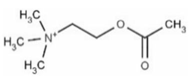	GPCR	MB	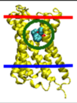
GABA$_A$ receptor	GABA$_A$-α1-6/β1-3/γ1-3/δ/ε/π/θ, ρ1-3	GABA	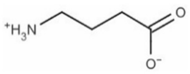	ion channel	EX	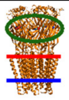
GABA$_B$ receptor	GABA$_{B1\text{-}2}$	GABA	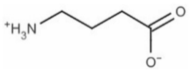	GPCR	EX	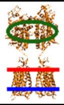
glycine receptor	GlyR-α1-4/β	glycine	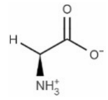	ion channel	EX	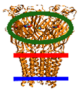
ionotropic glutamate receptor	GluA1-4, GluK1-5, GluN1, GluN2A-D, GluN3A-B	glutamate, glycine, serine, aspartate	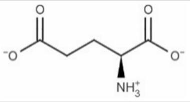	ion channel	EX	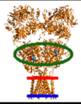
ionotropic serotonin receptor	5-HT3	serotonin	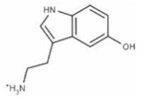	ion channel	EX	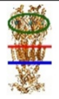
metabotropic glutamate receptor	mGluR1-8	glutamate	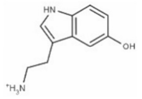	GPCR	EX	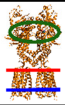
nicotinic acetylcholine receptor	nAch-α1-10/β1-4, D, E, G	acetylcholine	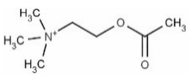	ion channel	EX	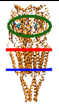

[1] A NT 2D structure is shown for each receptor type; however, adrenergic and ionotropic glutamate receptors have more than one activating NT. [2] GPCR is abbreviation for G protein-coupled receptor. Ion channel refers to ligand-gated ion channels. [3] The synaptic receptors have EXtracellular (EX) or Membrane-Buried (MB) ligand-binding sites are highlighted

for the 3D structures of the receptors (green circles). Both the extracellular (red) and intracellular (blue) surfaces are also indicated with lines.

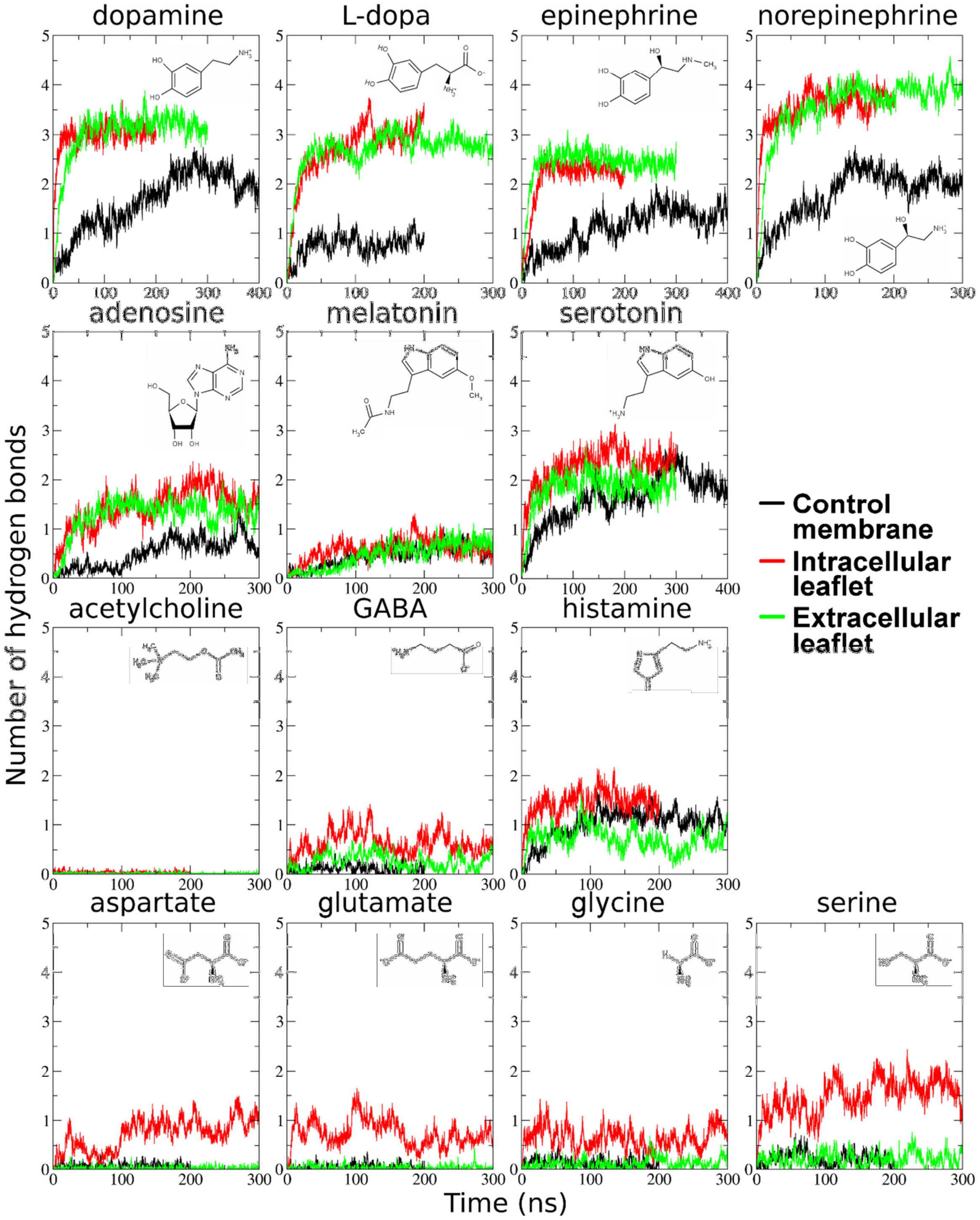

Figure S3. The amount of membrane hydrogen bonding per neurotransmitter. The NT-lipid bilayer H-bonding for the control membrane (DLPC; black; Figure S2), the intracellular leaflet (DLPC/DLPE/DLPS; red; Figure S2) and the extracellular leaflet (DOPC/SM/CHOL; green; Figure S2) models shown as a moving average of 10. The simulations were stopped as the curves plateaued or reached equilibrium stages; accordingly, the lengths of the simulation trajectories vary. The 2D structures of NTs (Figure S1) are included with the plotted curves.

Table S2. Membrane hydrogen bonding per neurotransmitter and average area per lipid values.

Neurotransmitter as 2D representation	Neurotransmitter + membrane model	Hydrogen bond[1]	Area per lipid[2]	Ligand-binding site position or the receptor[3]	
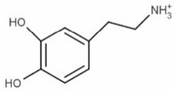	dopamine + control	2.03	0.64		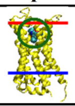
	dopamine + intracellular	3.05	0.62	MB	
	dopamine + extracellular	3.21	0.58		
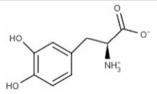	L-dopa + control	0.80	0.64		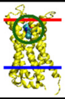
	L-dopa + intracellular	2.94	0.62	MB	
	L-dopa + extracellular	2.86	0.57		
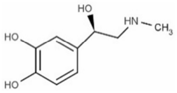	epinephrine + control	1.32	0.65		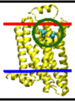
	epinephrine + intracellular	2.29	0.64	MB	
	epinephrine + extracellular	2.42	0.59		
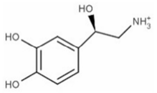	norepinephrine + control	2.08	0.63		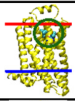
	norepinephrine + intracellular	3.68	0.61	MB	
	norepinephrine + extracellular	3.87	0.57		
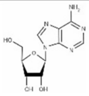	adenosine + control	0.75	0.64		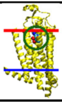
	adenosine + intracellular	1.71	0.63	MB	
	adenosine + extracellular	1.39	0.58		
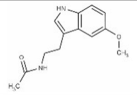	melatonin + control	0.58	0.67		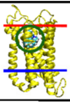
	melatonin + intracellular	0.69	0.64	MB	
	melatonin + extracellular	0.66	0.60		
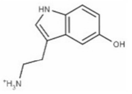	serotonin + control	2.05	0.65		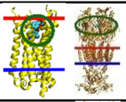
	serotonin + intracellular	2.44	0.62	MB/EX	
	serotonin + extracellular	1.92	0.58		
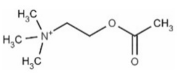	acetylcholine + control	0.02	0.63		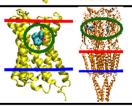
	acetylcholine + intracellular	0.03	0.62	MB/EX	
	acetylcholine + extracellular	0.02	0.57		
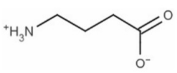	GABA + control	0.12	0.63		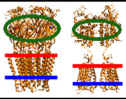
	GABA + intracellular	0.64	0.61	EX	
	GABA + extracellular	0.21	0.57		
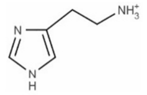	histamine + control	1.13	0.63		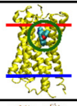
	histamine + intracellular	1.51	0.62	MB	
	histamine + extracellular	0.66	0.57		
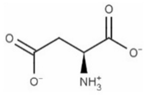	aspartate + control	0.06	0.62		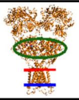
	aspartate + intracellular	0.92	0.61	EX	
	aspartate + extracellular	0.02	0.57		
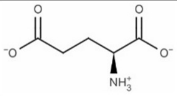	glutamate + control	0.06	0.62		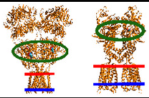
	glutamate + intracellular	0.63	0.61	EX	
	glutamate + extracellular	0.04	0.57		
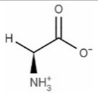	glycine + control	0.11	0.63		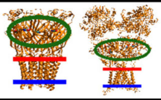
	glycine + intracellular	0.61	0.61	EX	
	glycine + extracellular	0.15	0.57		
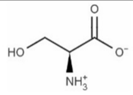	serine + control	0.23	0.62		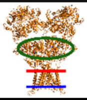
	serine + intracellular	1.68	0.60	EX	
	serine + extracellular	0.22	0.57		
	control membrane	-	0.63	-	
	intracellular leaflet	-	0.61	-	
	extracellular leaflet	-	0.57	-	

[1] The last 100 ns of equilibrated trajectory were used in the analysis. [2] A cutoff distance of 3.5 Å between the acceptor and donor was used as the H-bond criteria. [3] The receptors have Extracellular (EX) or Membrane-Buried (MB) binding sites. The ligand-binding sites are highlighted (green circles; Table S1) and both the extracellular (red) and intracellular (blue) surfaces are also indicated with lines.

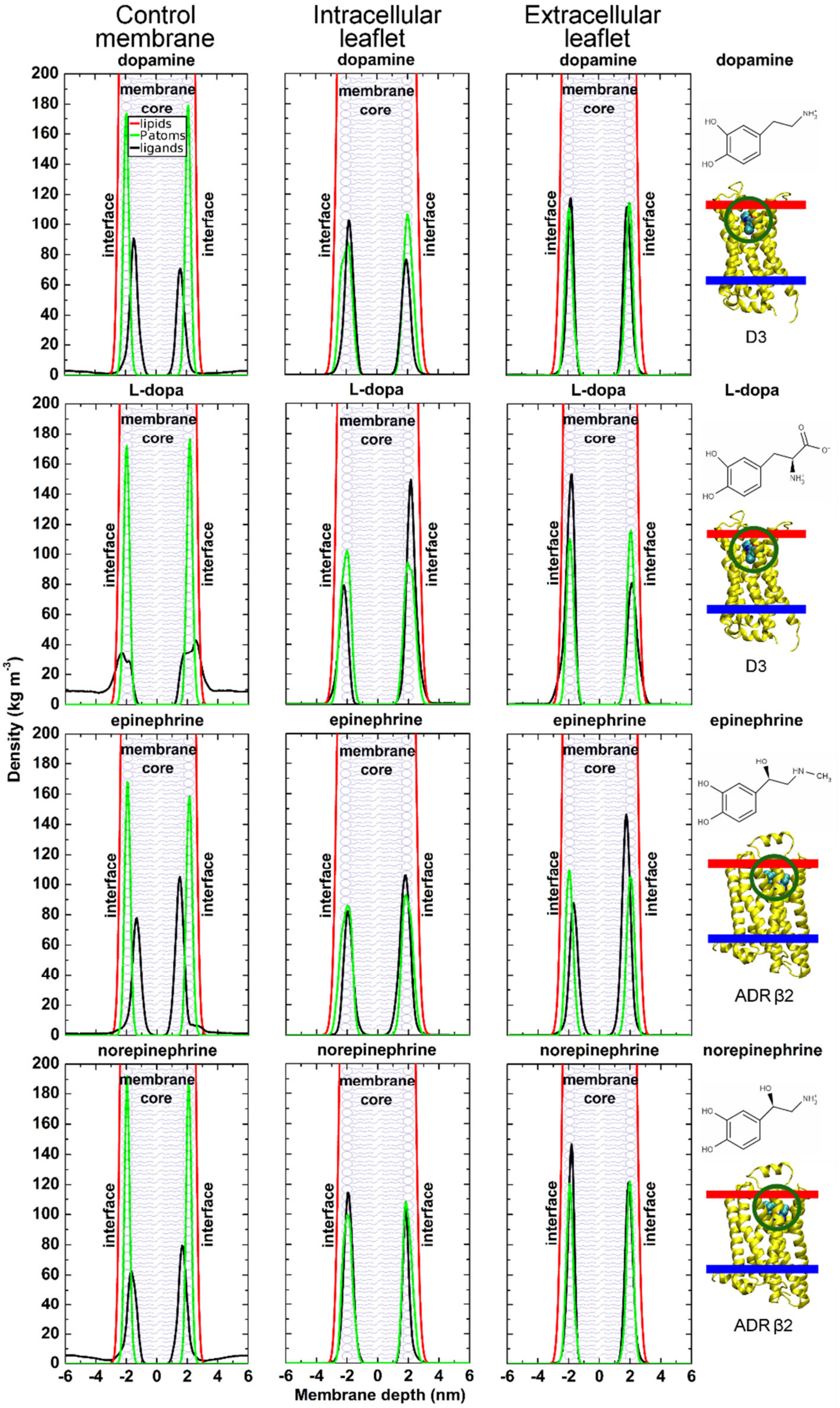

Figure S4. Neurotransmitter-membrane densities with group I molecules. The density curve was calculated for the NT (black line), lipids (red line), and phosphorus atoms (green line) of the lipid head groups. The ligand-binding sites of the catecholamine NTs are membrane-buried (green circles; Table S1). The ligands are shown as CPK models. Extracellular (red) and intracellular (blue) membrane sides are indicated with lines for the 3D receptor structures (Table S1) together with the 2D structures of the NTs on the right.

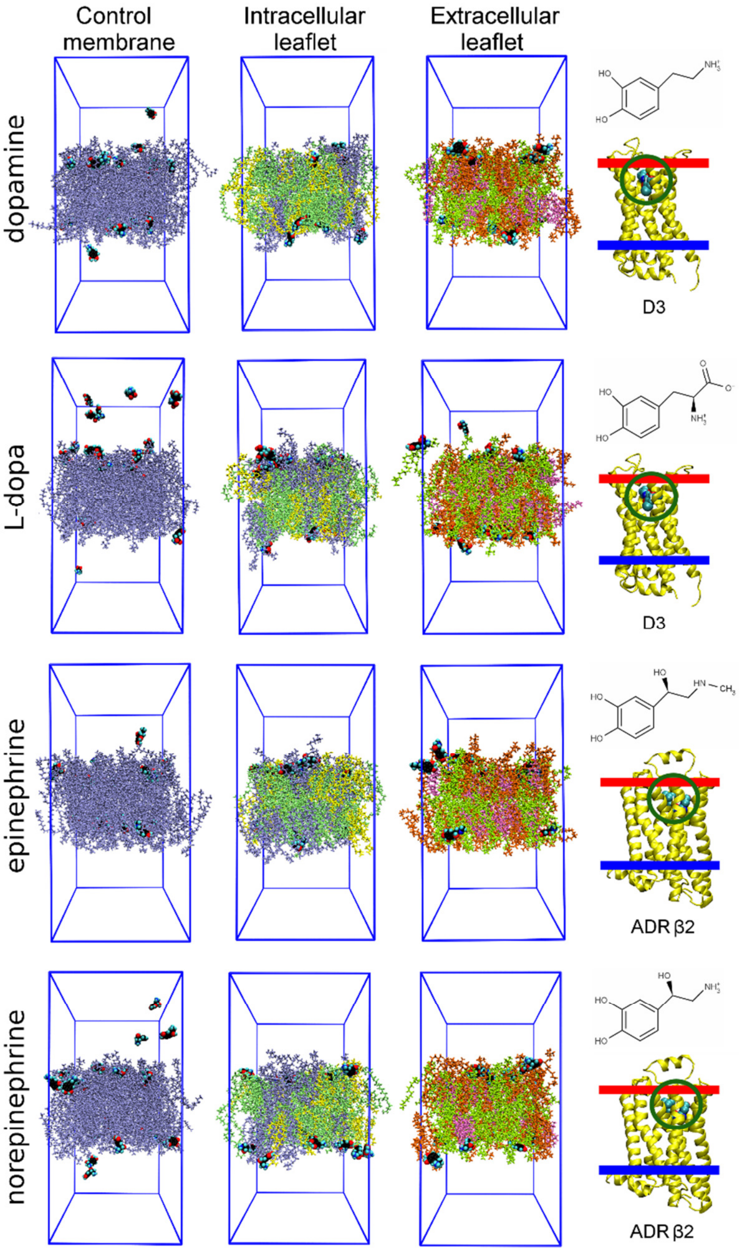

Figure S5. Neurotransmitter-membrane associations with group I molecules. The adherence of group I NTs (CPK models; Figure S1) to the membrane models is clearly visible when inspecting the last frames from the 200-300 ns simulation trajectories (see Figure S3 for individual running times). The control membrane contained DLPC (blue), the intracellular leaflet contained DLPC, DLPE (lime), and DLPS (yellow), the extracellular leaflet contained DOPC (orange), SM (orange), and CHOL (mauve). Lipids are shown as licorice models, while NTs are shown as CPK models. The ligand-binding sites of the catecholamine NTs are membrane-buried (green circles; Table S1). The boundaries of the simulation box is shown in blue. Extracellular (red) and intracellular (blue) membrane sides are indicated with lines for the 3D receptor structures together with the 2D structures of the NTs on the right.

Text 4. Predictive Power of the Octanol/Water Partition Coefficient.

Log P is the base-10 logarithmic measure of the octanol/water partition coefficient. It is used to estimate the lipophilicity of small chemical compounds in their un-ionized form. A high log P value suggests that a compound is lipophilic and more likely to partition into the cell membrane, while a low value indicates a preference for water. The NTs that do not adhere on the membrane surface in our simulations have lower log P values than the NTs that do (Table S3). The experimental log P values of membrane-binding NTs range from -2.39 to 1.60 (Table S4), while the values for membrane-nonbinding NTs range from -3.89 to -3.07 (Table S4). The experimental data was unavailable for desalted acetylcholine, but the predicted log P values of acetylcholine match the membrane binding behavior seen in the simulations. Significantly, the log P data allows the NTs to be grouped into membrane-binding (ALOGPS: -2.09 to 1.15 and ChemAxon:-2.3 to 1.42; Table S4) and membrane-nonbinding (-4.22 to -2.89 and -3.5 to -2.9; Table S4) categories without any overlap. Although there exist both membrane-buried and extracellular ligand-binding sites for serotonin and acetylcholine receptors (Table S1), both the log P values and the simulations suggested that the serotonin adheres and acetylcholine does not adhere on the extracellular leaflet (DOPC/SM/CHOL; Figure S2; Table S3). The log P value of histamine suggested similarly as the control membrane (DLPC; Figure S2) and intracellular leaflet (DLPC/DLPE/DLPS; Figure S2) simulations membrane-adherence (Table S3); however, the same effect was not seen in the extracellular leaflet simulation. As with our simulations, the division based on the log P values corresponds to the positioning of the receptors' ligand-binding sites in relation to the membrane (Figure 2; Table S3). The partition coefficients calculated based on the NT-membrane simulations (Tables 1 and S4) propose the same division into membrane-binding and membrane-nonbinding ranges with the control membrane (0.20 to 1.31 vs. -0.66 to -0.39; Table S4), the intracellular leaflet (0.78 to 3.61 vs. -0.27 to 0.28; Table S4) and the extracellular leaflet (-0.14 to 3.67 vs. -0.64 to 0.34; Table S4) membrane models. The slight overlap of the extracellular leaflet ranges is due to the fact that histamine prefers the water phase over the extracellular leaflet in our simulation (discussed in more detail below).

Table S3. Predicted and experimental water/octanol partition coefficients.

Neurotransmitter as 2D representation	Neurotransmitter	Log P ChemAxon[1]	Log P ALOGPS[2]	Log P Experimental[3]	Ligand-binding site position of the receptor[4]
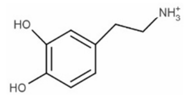	dopamine	0.03	-0.40	-0.98	MB 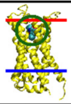
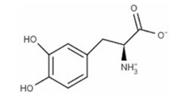	L-dopa	-1.79	-2.30	-2.39	MB 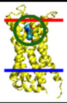
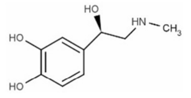	epinephrine	-0.43	-0.82	-1.37	MB 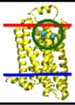
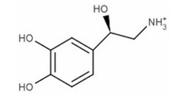	norepinephrine	-0.43	-1.40	-1.24	MB 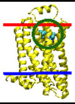
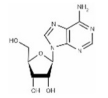	adenosine	-2.09	1.20	-1.05	MB 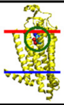
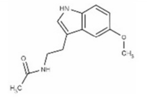	melatonin	1.15	1.42	1.60	MB 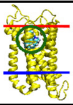
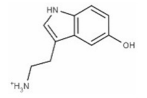	serotonin	0.48	0.56	0.21	MB/EX 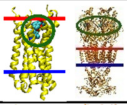
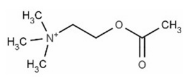	acetylcholine	-4.22	-2.90	-	MB/EX 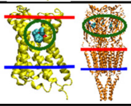
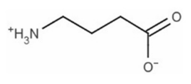	GABA	-2.89	-3.00	-3.37	EX 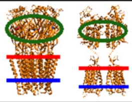
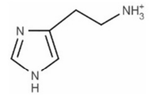	histamine	-0.70	-0.69	-0.70	MB 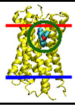
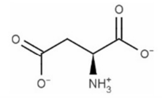	aspartate	-3.50	-3.50	-3.89	EX 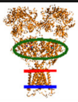
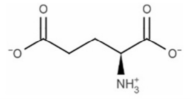	glutamate	-3.24	-3.50	-3.69	EX 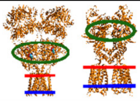
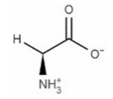	glycine	-3.41	-3.30	-3.21	EX 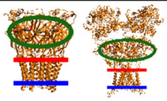
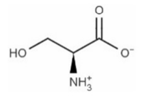	serine	-3.89	-3.40	-3.07	EX 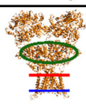

[1] Predicted log P values acquired from ChemAxon (http://www.chemicalize.org). [2] Predicted log P values acquired from ALOGPS (http://www.vcclab.org/lab/alogps/). [3] The experimental log P values were acquired from DrugBank (http://www.drugbank.ca). The experimental log P values for GABA, histamine, and serotonin were acquired from LOGKOW (http://logkow.cisti.nrc.ca/logkow/index.jsp). The receptors have EXtracellular or Membrane-Buried binding

sites. The ligand-binding sites are highlighted (green circles; Table S1) and both the extracellular (red) and intracellular (blue) surfaces are also indicated with lines.

Table S4. The partition coefficient ranges of neurotransmitters follow ligand-binding site positioning.

Method + Solvents	Extracellular ligand-binding site[5]	Membrane-buried ligand-binding site[5]
MD + water/control membrane[1]	-0.66 to -0.39	0.20 to 1.31
MD + water/intracellular leaflet[1]	-0.27 to 0.28	0.78 to 3.61
MD + water/extracellular leaflet[1]	-0.64 to 0.34	-0.14 to 3.67
ChemAxon + water/octanol[2]	-3.50 to -2.90	-2.09 to 1.15
ALOGPS + water/octanol[3]	-4.22 to -2.89	-2.30 to 1.42
Experimental + water/octanol[4]	-3.89 to -3.07	-2.39 to 1.60

[1] LOG partition coefficient = LOG(NT at membrene/NT in water). The NT was considered bound at the membrane if it resided ±1.0 nm from the head group nitrogen atom density average of either membrane leaflet. [2] First computational 10-base logarithmic water/octanol partition coefficient or log P value by ChemAxon (http://www.chemicalize.org) and [3] second computational log P value by ALOGPS (http://www.vcclab.org/lab/alogps/). [4] Experimental log P value from DrugBank (http://www.drugbank.ca) or from LOGKOW (http://logkow.cisti.nrc.ca/logkow/index.jsp). [5] Each receptor has either extracellular or membrane-buried ligand-binding site (Table S1).

Text 5. Serotonin and Melatonin Attach Strongly to the Membrane.

Group II molecules, which include adenosine, melatonin and serotonin, were grouped together as they have roughly similar double ring system as a scaffold (Figure S1). Melatonin and serotonin have an indole ring and adenosine has a nitrogen-rich purine ring; however, the other constituents or side chains of the NTs are noticeably different. Despite these structural differences each of these molecules have similar tendency to form H-bonds with the membrane models in the simulations (Figure S3; Table S2). The group II NTs form the highest amount of H-bonds with the intracellular leaflet (DLPC/DLPE/DLPS; Figure S2) while the less polar control membrane (DLPC; Figure S2) induced clearly lesser amount of H-bonding. Of the three compounds serotonin H-bonded the most with the membrane models while adenosine bonding was the weakest (Table S2). Serotonin was able to form one H-bond more than melatonin because it has an extra H-bond donor group attached to its ring system if compared to the bulkier melatonin. The density profiles indicate that both melatonin and serotonin formed tight associations with the studied membrane models (Figure S6; Table 1). The NT-membrane association was stronger with the extracellular leaflet (DOPC/SM/CHOL; Figure S2) and intracellular leaflet models than with the control membrane (Figure S6; Table 1). Similarly as catecholamines (see above) serotonin and melatonin were buried relatively deep inside the control membrane. This behavior is explained by the fact that these molecules contain a hydrophobic ring system and H-bond donor groups. With the control membrane melatonin could bury itself deeper into the membrane than serotonin (Figure S7). Bulky NT adenosine was unable to form close association with any of the bilayers; this was especially the case with the control membrane (Figure S6). Reflecting the strong H-bonding capacity of serotonin (Figure S3), all serotonin molecules were bound to the membrane model surfaces in the last simulation frames (Figure S7). The ligand-binding site positioning of serotonin receptors is complicated – there exist both extracellular and membrane-buried binding sites depending on the receptor type (Table S1; Figure S6); however, both NT-membrane simulations (Table 1) and the log P value (Table S3) suggest that serotonin adheres preferentially on synaptic membrane. The final positioning of melatonin was equally tight with the control membrane and the

intracellular leaflet. On the other hand, adenosine molecules were more dispersed with membrane models than serotonin and melatonin as already suggested by the H-bonding and density profiles (Figures S3 and S6; Tables S2 and 1). The double ring systems made it possible that adenosine, serotonin and melatonin could stack together more than other NTs simulated in this study; but the NT clusters were not enduring on the membrane surface (Figure S7).

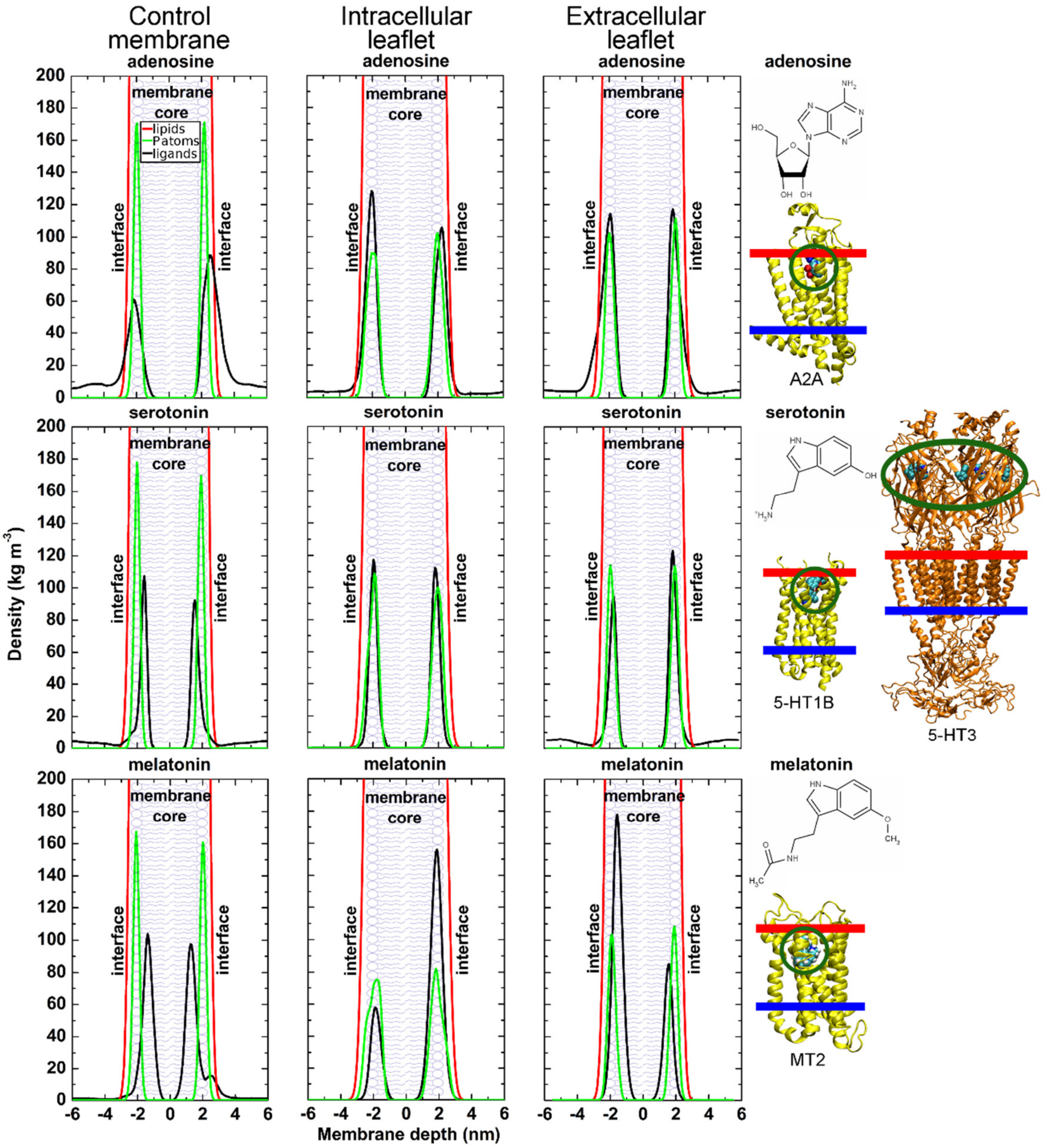

Figure S6. Neurotransmitter-membrane densities with group II molecules. The ligand-binding sites of the NTs are membrane-buried (green circles); however, for serotonin there exist a receptor type (5-HT3) that have extracellular binding sites (Table S1). For details see Figure S4.

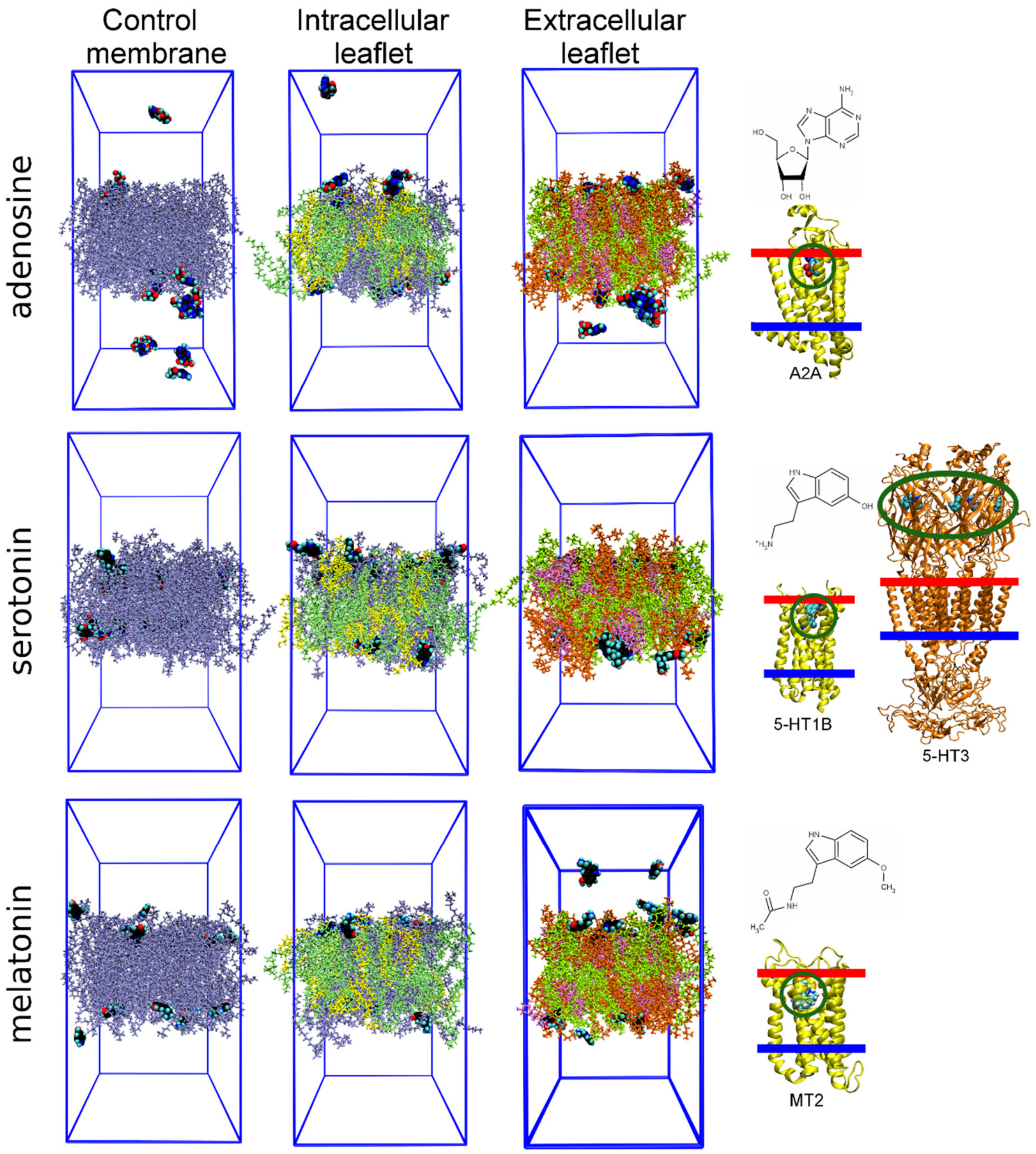

Figure S7. Neurotransmitter-membrane association with group II molecules. The ligand-binding sites of the NTs are membrane-buried (green circles); however, for serotonin there exist a receptor type (5-HT3) that has extracellular binding sites (Table S1). For details see Figure S5.

Text 6. Histamine-Membrane Association at an Intermediate level.
Group III NTs acetylcholine, γ-aminobutyric acid (GABA), and histamine form a miscellaneous group of relatively small molecules (Figure S1). Acetylcholine and histamine have in common a net positive charge. While GABA also has a positively charged group, it does not have a net charge. Despite their structural dissimilarities the molecules are alike in their inability to form lasting H-bonds with the lipid bilayers (Figure S3; Table S2). Histamine is able to form at least one H-bond while acetylcholine does not H-bond at all and GABA is in the threshold of forming a single H-bond similarly as melatonin (see above). The highest level of H-bonding was produced with the intracellular leaflet (DLPC/DLPE/DLPS; Figure S2) while the extracellular leaflet (DOPC/SM/CHOL; Figure S2) produced the second highest H-bonding score (except with histamine). The density profiles indicate that Group III NTs do not adhere strongly on the control membrane (DLPC; Figure S2) or the extracellular leaflet (Figure S8; Table 1). Because all three NTs have positively charged group, they formed the strongest association with the intracellular leaflet containing negatively charged DLPS lipids (Table 1). One could argue that the lipid environment of histamine receptors should contain negatively charged residues such as glycolipids or DLPS, because the receptor's ligand binding site is membrane-buried (Table S1; Figure S8) and it was the only NT of the group to adhere on the intracellular leaflet but not on the extracellular leaflet. Histamine also preferred the less polar control membrane over the water phase unlike acetylcholine and GABA. The ability of histamine to enter deep into the control membrane is conveyed by its hydrophobic ring. The strong preference of acetylcholine towards the intracellular leaflet might explain the existence of two very different kind of acetylcholine receptors (Table S1; Figure S8). On one hand, acetylcholine is not expected to adhere on the synaptic membrane as is seen with the extracellular leaflet and control simulations (membrane-independent mechanism; Figure 3a), because nicotinic acetylcholine receptors have extracellular ligand-binding sites (Table S1; Figure S8). On the other hand, the adherence of acetylcholine on the more negatively-charged intracellular leaflet (membrane-dependent mechanism; Figure 3b) is in line with the fact that muscarinic acetylcholine receptors have membrane-buried ligand-binding sites (Table S1; Figure S8). Based on the density profiles (Figure S8) and simulation end stages (Figure S9) it is clear that histamine is interacting moderately strongly with the intracellular leaflet.

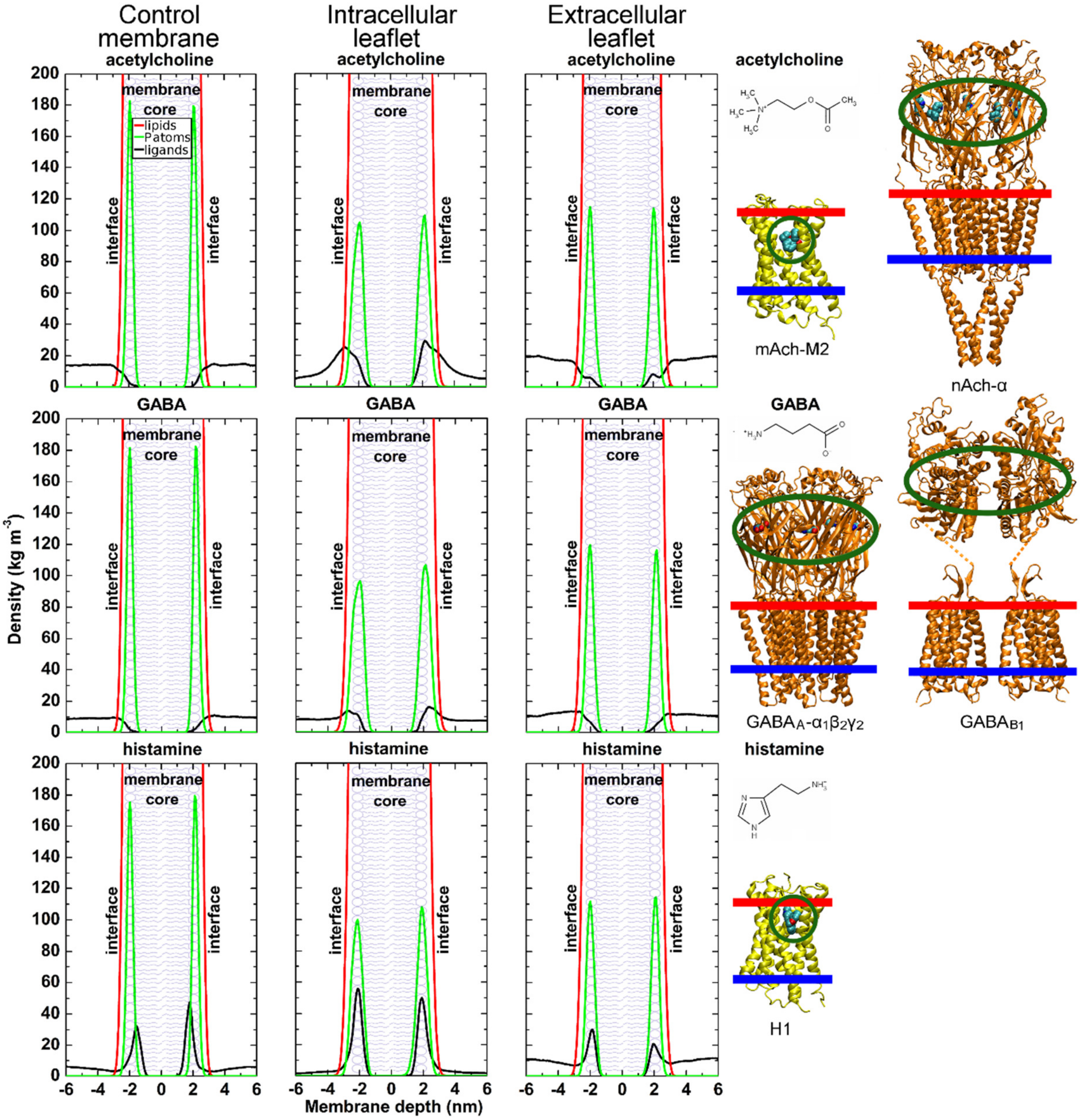

Figure S8. Neurotransmitter-membrane densities with group III molecules. The ligand-binding sites of $GABA_A$ and $GABA_B$ receptors are extracellular. The binding site for histamine is membrane-buried. With acetylcholine there exist membrane-buried (mAch-M2) and extracellular (nAch-α) ligand-binding sites (green circles). For details see Figure S4.

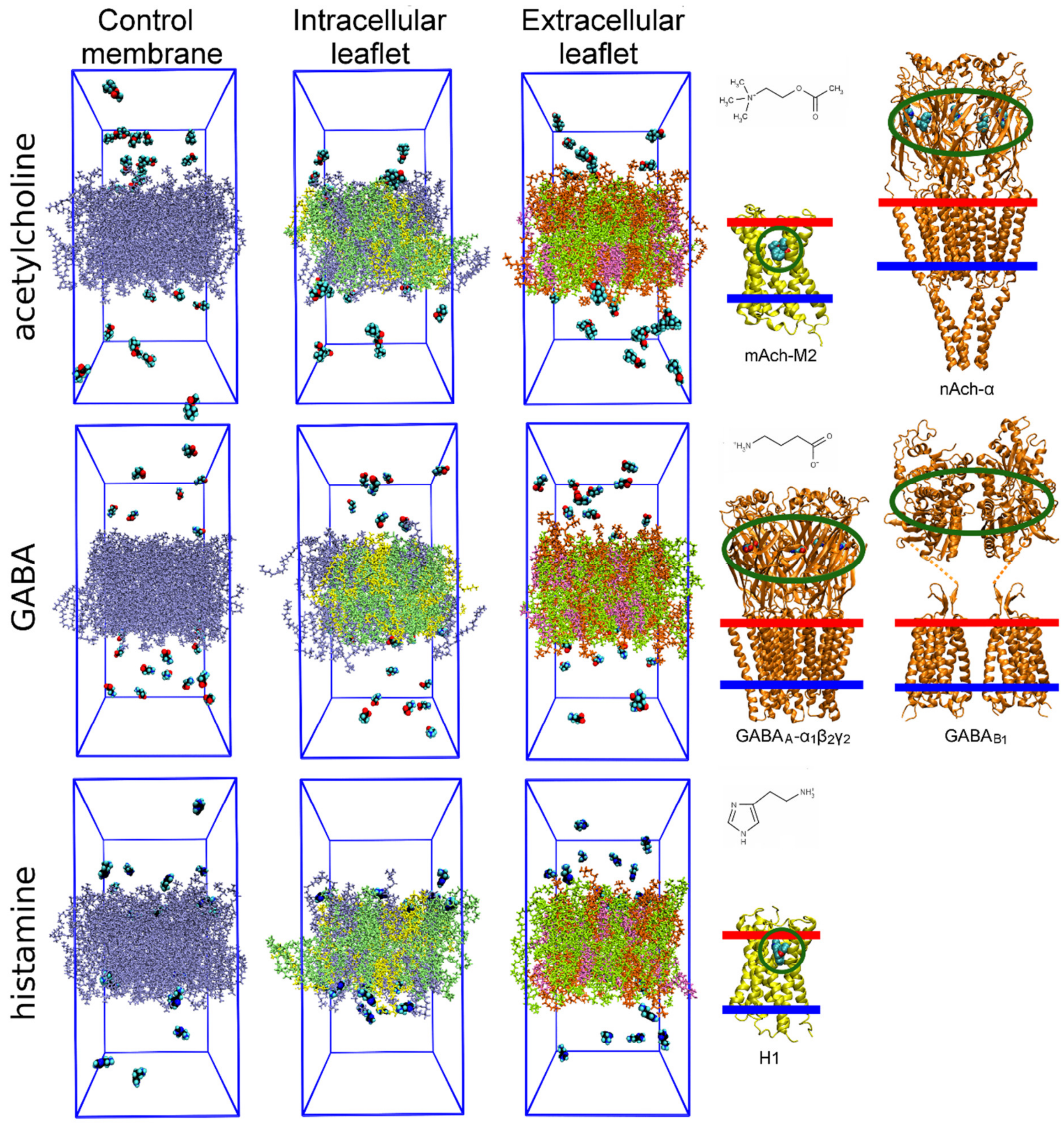

Figure S9. Neurotransmitter-membrane association with group III molecules. The ligand-binding sites of $GABA_A$ and $GABA_B$ receptors are extracellular. The $GABA_B$ receptor, whose function is G protein-coupled, is not shown here (Table S1; Figure S2). The binding site for histamine is membrane-buried. With acetylcholine there exist membrane-buried (mAch-M2) and extracellular (nAch-α) ligand-binding sites (green circles). For details see Figure S5.

Text 7. Amino Acids Do Not Bind to the Membrane Surfaces.
Group IV NTs include amino acids aspartate, glutamate, glycine and serine (Figure S1). The NTs have in common their relatively small size and the basic amino acid backbone including charged amine (NH_3^+) and carboxylate (COO^-) groups. Despite the H-bonding capacity of the amino acids, they did not have a strong tendency to form H-bonds with the membrane models (Figure S3; Table S2). The amino acids formed more H-bonds with the intracellular leaflet (DLPC/DLPE/DLPS; Figure S2) membrane than with the other two membranes. Interestingly, the amino acids formed more H-bonds with the control membrane (DLPC; Figure S2) than with the extracellular leaflet (DOPC/SM/CHOL; Figure S2) membrane – a result that reminds the H-bonding of histidine derivative histamine (see above). Furthermore, serine was able to form more H-bonds than other amino acids. The level of H-bonding between glycine and the control membrane and the extracellular leaflet was relatively high; however, both aspartate and glutamate produced higher amount of H-bonding with the intracellular leaflet than serine. Although aspartate and glutamate produced almost similar H-bonding with the control membrane and the extracellular leaflet, it is worth noting that aspartate formed more H-bonds with the intracellular leaflet than similar amino acid glutamate. The density profiles indicate that none of the amino acids adhered strongly on the membrane surfaces (Figure S10). The only noticeable membrane adherence took place at the intracellular leaflet. Serine even slightly preferred it (Table 1), although the ligand-binding sites of NMDA receptors are extracellular (Table S1). Again, the preference to aggregate on top of the intracellular leaflet can be explained by the negatively charged DLPS and the positively charged amine groups of the amino acids. However, when inspecting the last simulation frames, it is evident that none of the amino acids really adhered strongly on the membrane surfaces (Figure S11).

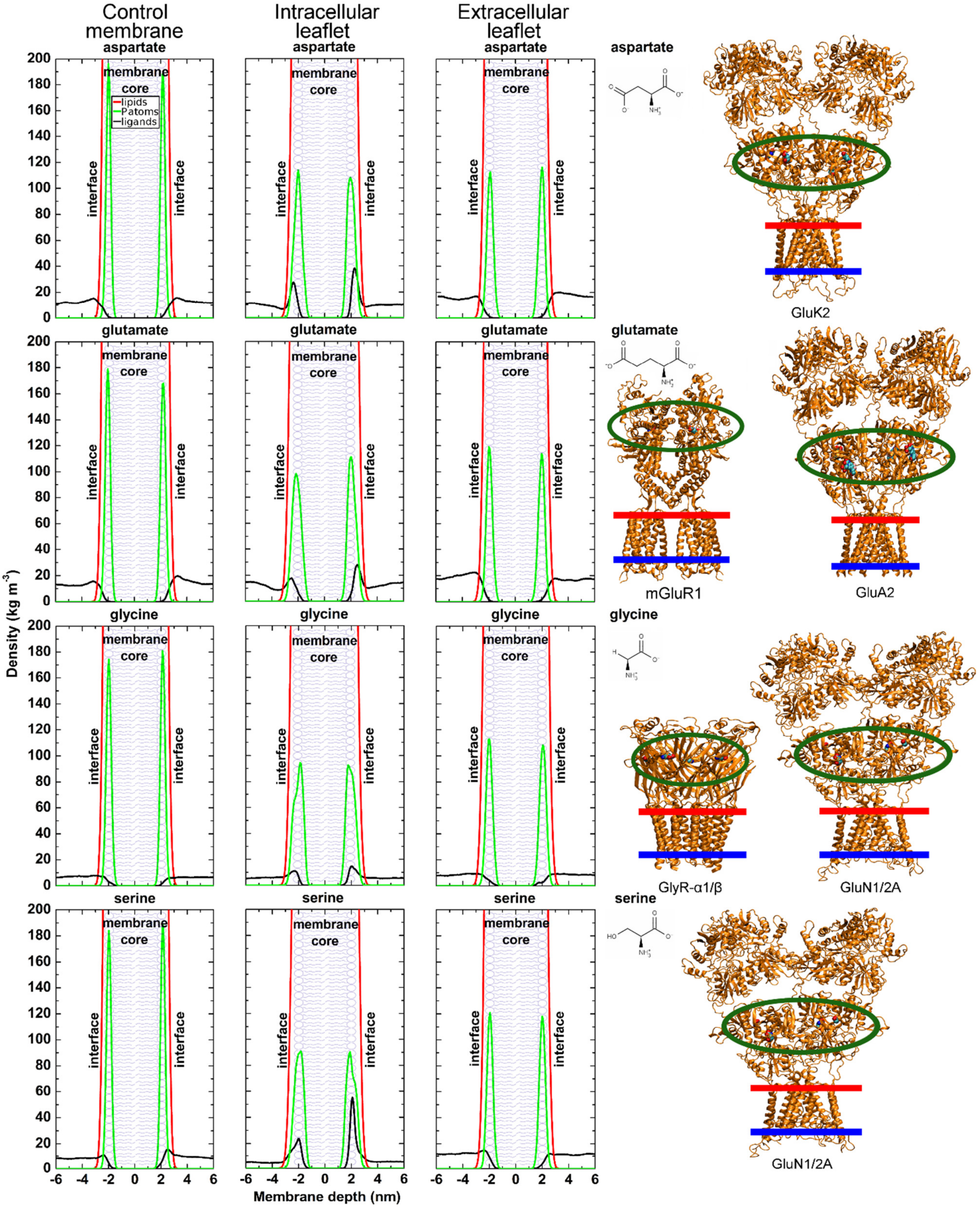

Figure S10. Neurotransmitter-membrane densities with group IV molecules. The ligand-binding sites of amino acid NTs are extracellular. For details see Figure S4.

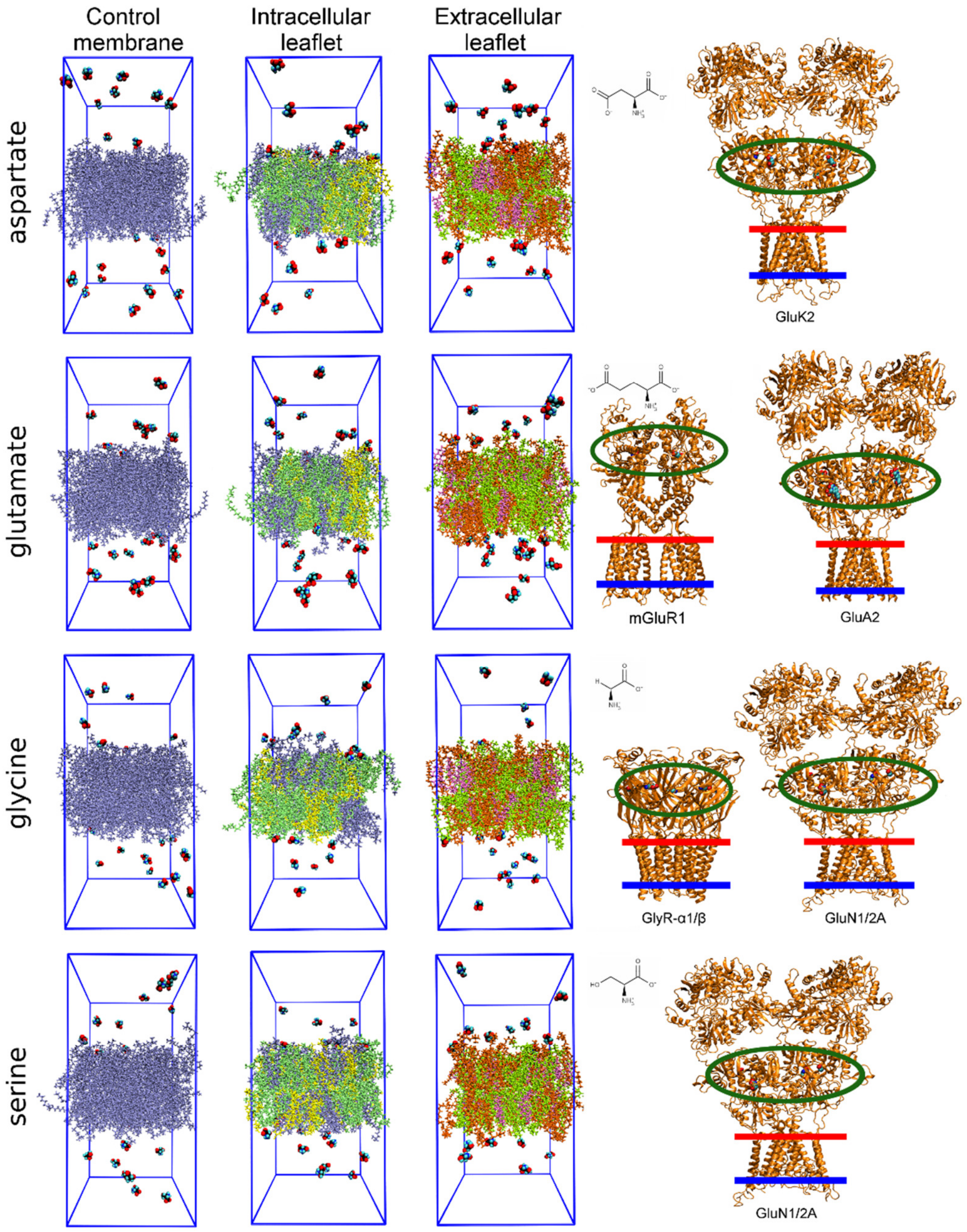

Figure S11. Neurotransmitter-membrane association with group IV molecules. The ligand-binding sites of amino acid NTs are extracellular. For details see Figure S5.

Text 8. Free Energy of Profiles.

The negatively charged glutamate clearly preferred the water phase over the membrane models based on the free energy calculations (Figure 3). In fact, the energy minimum of glutamate was positioned at the membrane surface only with the intracellular leaflet (DLPC/DLPE/DLPS; Figure S2) model. The energy difference between the bulk water and the intracellular leaflet, however, was tiny suggesting that glutamate is not adhering strongly on the surface (Table S5). This result could already be seen from H-bonding, density profile and the final snap shot (Figures S3, S10 and S11; Tables S2 and 1) of the classical MD simulations. On the other hand, norepinephrine, dopamine, and serotonin favored consistently the membrane surfaces (~2.5-2 nm) over the bulk water. The lowest energies were reported for the extracellular leaflet (DOPC/SM/CHOL; Figure S2) and generally the intracellular leaflet produced the second lowest energy minimum (Figure 3). These results correspond closely to the H-bonding and density profile values of these NTs (Tables S2 and 1). Notably, serotonin favored slightly the control membrane (DLPC; Figure S2) over the intracellular leaflet. Due to the favorable hydrophobic interactions serotonin could enter deep into the control membrane and, moreover, it occasionally formed as many H-bonds with the control membrane as with the intracellular leaflet (Figure S3). Both synaptic membrane models produced larger energy differences than the control membrane (Table S5). With dopamine and serotonin the differences were largest for the extracellular leaflet but with norepinephrine the biggest difference was reported for the intracellular leaflet. Notably all of the energy differences were lower for the control membrane, which suggests that the aggregation on this surface would be slower than on the two synaptic membrane models. This predicted slowness is reflected on the amount of simulation time that was needed to level out the H-bonding between the NTs and the control membrane (Figure S3). Relatively low energy differences were also seen with serotonin-membrane systems. This weak interaction was again reflected on the simulation time needed to equilibrate serotonin-membrane H-bonding (Figure S3). The positioning of the energy minima of free energy profiles of the NTs at the membrane is in perfect correlation with the known ligand-binding site positioning of their receptors (Table S1). Norepinephrine, epinephrine and serotonin, that have membrane-buried ligand-binding sites, preferred the water-membrane interface while only the negatively-charged glutamate, whose receptors' ligand-binding sites are extracellular, had its energy minimum in the bulk water (Figure 3; Table S5).

Table S5. Free energy difference between bulk water and the water-membrane interface for neurotransmitters.

**Neurotransmitter as 2D representation**	**Neurotransmitter + membrane model**	**EΔ (kJ mol-1)[1]**	**Ligand-binding site position of the receptor[2]**	
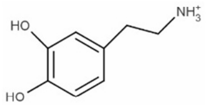	dopamine + control	13.78	MB	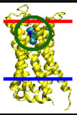
	dopamine + intracellular	21.15		
	dopamine + extracellular	***21.51***		
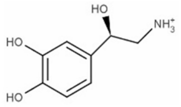	norepinephrine + control	11.65	MB	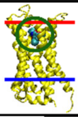
	norepinephrine + intracellular	***22.03***		
	norepinephrine + extracellular	21.02		
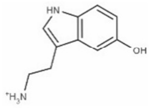	serotonin + control	11.56	MB/EX	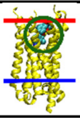 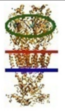
	serotonin + intracellular	13.24		
	serotonin + extracellular	***14.38***		
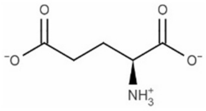	glutamate + control	-	EX	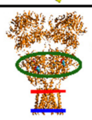 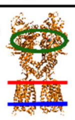
	glutamate + intracellular	***3.36***		
	glutamate + extracellular	-		

[1] EΔ is the free energy difference between bulk water and the water-membrane interface location. The biggest energy differences between the bulk water and membrane surface for each NT are shown in bold and underlined. The receptors have EXtracellular or Membrane-Buried binding sites. The ligand-binding sites are highlighted (green circles; Table S1) and both the extracellular (red) and intracellular (blue) surfaces are also indicated with lines.

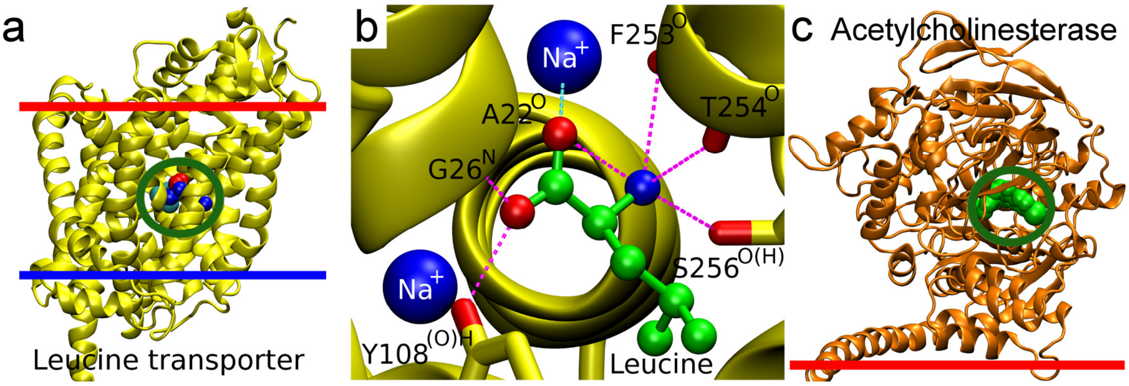

Figure S12. Role of membrane and ions when turning down the neurotransmitter-mediated signal. a) The binding site (green circle) of leucine (CPK model) inside its bacterial transporter (yellow cartoon) is membrane-buried. The leucine transporter is a bacterial homolog of mammalian NT transporters; accordingly, based on sequence comparison the NT binding mode likely reminds the one reported for leucine (23). b) The bound leucine (ball-and-stick model with green backbone) not only forms hydrogen bonds (dotted magenta lines) with the protein residues but also a direct ionic bond (cyan dotted line) with one sodium ion as well. c) The binding site of acetylcholine (green CPK model) is extracellular in human acetylcholinesterase structure (orange cartoon). d) The ligand-binding sites (green circles) of monoamine oxidase B dimer are not membrane-buried but cytoplasmic (orange cartoon), because the protein is monotopically inserted into mitochondrial outer membrane. c. The monoamine NTs have been suggested to enter the enzyme's active sites, which are adjacent to the co-factor flavin adenine nucleotides (CPK models with cyan backbone), via hydrophobic entrance cavities originating from close to the membrane surface. The routes into the binding sites through the entrance cavities are indicated roughly with yellow arrows. The extracellular and intracellular surfaces of cell membrane are shown with red and blue lines, respectively. Similarly, the intermembrane surface (magenta) and cytoplasmic surface (cyan) of mitochondrial outer membrane are shown with lines.

Table S6. Predicted water/octanol partition coefficients for active DUD compounds.

Neurotransmitter as 2D representation	Neurotransmitter	Neurotransmitter binding protein[1]	AVE Log P[2]	MAX Log P[2]	MIN Log P[2]	MED Log P[2]	Neurotransmitter Log P[3]	Ligand-binding site position of the protein[4]
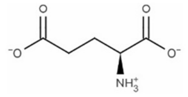	glutamate	GluA2 receptor	0.66	5.81	-3.91	-0.75	-3.24	EX 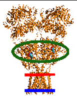
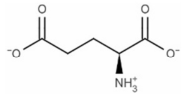	glutamate	GluK1 receptor	-0.73	3.31	-3.91	1.75	-3.24	EX 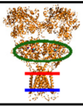
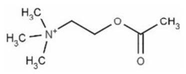	acetylcholine	Acetylcholinesterase	5.22	11.8	-1.96	4.98	-4.22	EX 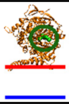
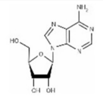	adenosine	A_{2A} receptor	2.45	7.66	-2.83	3.39	-2.09	MB 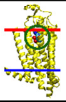
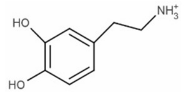	dopamine	D_3 receptor	4.78	9.1	-0.98	4.98	0.03	MB 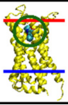
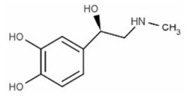	epinephrine	ADR-β_2 receptor	3.15	6.58	-2.3	2.53	-0.43	MB 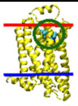

[1] Neurotransmitter-binding proteins, including selected synaptic receptors and acetylcholinesterase, for which there exist active molecule sets in the Directory of Useful Decoys (DUD; http://dud.docking.org/) are shown. [2] Log P values predicted using QikProp in MAESTRO10.0 (Schrödinger, LLC, New York, NY, 2014) for the DUD actives. [3] Predicted log P values acquired by ALOGPS (http://www.vcclab.org/lab/alogps/) for the neurotransmitters. [4] The receptors have EXtracellular or Membrane-Buried binding sites. The ligand-binding sites are highlighted (green circles; Table S1) and both the extracellular (red) and intracellular (blue) surfaces are also indicated with lines.

Text 9. Supplementary References.

1. Hanwell MD, Curtis DE, Lonie DC, Vandermeersch T, Zurek E, Hutchison GR (2012): Avogadro: an advanced semantic chemical editor, visualization, and analysis platform. *J Cheminform* 4: 17.
2. Dupradeau F, Pigache A, Zaffran T, Savineau C, Lelong R, Grivel N*, et al.* (2010): The R.E.D. tools: advances in RESP and ESP charge derivation and force field library building. *Phys. Chem. Chem. Phys.* 12: 7821.
3. Jorgensen WL, Maxwell DS, Tirado-Rives J (1996): Development and Testing of the OPLS All-Atom Force Field on Conformational Energetics and Properties of Organic Liquids. *J. Am. Chem. Soc.* 118: 11225–11236.
4. Kaminski GA, Friesner RA, Tirado-Rives J, Jorgensen WL (2001): Evaluation and Reparametrization of the OPLS-AA Force Field for Proteins via Comparison with Accurate Quantum Chemical Calculations on Peptides †. *J. Phys. Chem. B* 105: 6474–6487.
5. Takamori S, Holt M, Stenius K, Lemke EA, Grønborg M, Riedel D*, et al.* (2006): Molecular anatomy of a trafficking organelle. *Cell* 127: 831–846.
6. Lehtonen JV, Still D, Rantanen V, Ekholm J, Björklund D, Iftikhar Z*, et al.* (2004): BODIL: a molecular modeling environment for structure-function analysis and drug design. *J. Comput. Aided Mol. Des.* 18: 401–419.
7. Jorgensen WL, Chandrasekhar J, Madura JD, Impey RW, Klein ML (1983): Comparison of simple potential functions for simulating liquid water. *J. Chem. Phys.* 79: 926.
8. Hess B, Kutzner C, van der Spoel D, Lindahl E (2008): GROMACS 4: Algorithms for Highly Efficient, Load-Balanced, and Scalable Molecular Simulation. *J. Chem. Theory Comput.* 4: 435–447.
9. Maciejewski A, Pasenkiewicz-Gierula M, Cramariuc O, Vattulainen I, Rog T (2014): Refined OPLS all-atom force field for saturated phosphatidylcholine bilayers at full hydration. *J Phys Chem B* 118: 4571–4581.
10. Hess B, Bekker H, Berendsen HJC, Fraaije JGEM (1997): LINCS: A linear constraint solver for molecular simulations. *J. Comput. Chem.* 18: 1463–1472.
11. Nosé S (1984): A unified formulation of the constant temperature molecular dynamics methods. *J. Chem. Phys.* 81: 511.
12. Parrinello M (1981): Polymorphic transitions in single crystals: A new molecular dynamics method. *J. Appl. Phys.* 52: 7182.
13. Essmann U, Perera L, Berkowitz ML, Darden T, Lee H, Pedersen LG (1995): A smooth particle mesh Ewald method. *J. Chem. Phys.* 103: 8577.
14. Hub JS, Groot BL de, van der Spoel D (2010): g_wham—A Free Weighted Histogram Analysis Implementation Including Robust Error and Autocorrelation Estimates. *J. Chem. Theory Comput.* 6: 3713–3720.
15. Humphrey W, Dalke A, Schulten K (1996): VMD: visual molecular dynamics. *J Mol Graph* 14: 33-8, 27-8.
16. Berman HM, Westbrook J, Feng Z, Gilliland G, Bhat TN, Weissig H*, et al.* (2000): The Protein Data Bank. *Nucleic Acids Res.* 28: 235–242.
17. Lebon G, Warne T, Edwards PC, Bennett K, Langmead CJ, Leslie AGW*, et al.* (2011): Agonist-bound adenosine A2A receptor structures reveal common features of GPCR activation. *Nature* 474: 521–525.
18. Chien EYT, Liu W, Zhao Q, Katritch V, Won Han G, Hanson MA*, et al.* (2010): Structure of the Human Dopamine D3 Receptor in Complex with a D2/D3 Selective Antagonist. *Science* 330: 1091–1095.

19. Hanson MA, Cherezov V, Griffith MT, Roth CB, Jaakola V, Chien EY*, et al.* (2008): A Specific Cholesterol Binding Site Is Established by the 2.8 Å Structure of the Human β2-Adrenergic Receptor. *Structure* 16: 897–905.
20. Shimamura T, Shiroishi M, Weyand S, Tsujimoto H, Winter G, Katritch V*, et al.* (2011): Structure of the human histamine H1 receptor complex with doxepin. *Nature* 475: 65–70.
21. Wang C, Jiang Y, Ma J, Wu H, Wacker D, Katritch V*, et al.* (2013): Structural Basis for Molecular Recognition at Serotonin Receptors. *Science* 340: 610–614.
22. Sobolevsky AI, Rosconi MP, Gouaux E (2009): X-ray structure, symmetry and mechanism of an AMPA-subtype glutamate receptor. *Nature* 462: 745–756.
23. Yamashita A, Singh SK, Kawate T, Jin Y, Gouaux E (2005): Crystal structure of a bacterial homologue of Na+/Cl--dependent neurotransmitter transporters. *Nature* 437: 215–223.
24. Nachon F, Carletti E, Ronco C, Trovaslet M, Nicolet Y, Jean L*, et al.* (2013): *Human acetylcholinesterase in complex with huprine W and fasciculin 2*.
25. Bonivento D, Milczek EM, McDonald GR, Binda C, Holt A, Edmondson DE*, et al.* (2010): *human monoamine oxidase B in complex with 2-(2-benzofuranyl)-2- imidazoline*.
26. Haga K, Kruse AC, Asada H, Yurugi-Kobayashi T, Shiroishi M, Zhang C*, et al.* (2012): *Structure of the human M2 muscarinic acetylcholine receptor bound to an antagonist*.
27. Unwin N (2005): Refined Structure of the Nicotinic Acetylcholine Receptor at 4Å Resolution. *Journal of Molecular Biology* 346: 967–989.
28. Celie PHN, van Rossum-Fikkert SE, van Dijk WJ, Brejc K, Smit AB, Sixma TK (2004): Nicotine and carbamylcholine binding to nicotinic acetylcholine receptors as studied in AChBP crystal structures. *Neuron* 41: 907–914.
29. Lomize MA, Lomize AL, Pogozheva ID, Mosberg HI (2006): OPM: orientations of proteins in membranes database. *Bioinformatics* 22: 623–625.
30. Pala D, Beuming T, Sherman W, Lodola A, Rivara S, Mor M (2013): Structure-Based Virtual Screening of MT 2 Melatonin Receptor: Influence of Template Choice and Structural Refinement. *J. Chem. Inf. Model.* 53: 821–835.
31. Kiefer F, Arnold K, Künzli M, Bordoli L, Schwede T (2009): The SWISS-MODEL Repository and associated resources. *Nucleic Acids Res.* 37: D387-92.
32. Kopp J, Schwede T (2004): The SWISS-MODEL Repository of annotated three-dimensional protein structure homology models. *Nucleic Acids Res.* 32: D230-4.
33. Hilf R, Dutzler R (2008): *X-ray structure of a pentameric ligand gated ion channel from Erwinia chrysanthemi (ELIC)*.
34. Bergmann R, Kongsbak K, Sørensen PL, Sander T, Balle T, Permyakov EA (2013): A Unified Model of the GABAA Receptor Comprising Agonist and Benzodiazepine Binding Sites. *PLoS ONE* 8: e52323.
35. Hibbs RE, Gouaux E (2011): *C. elegans glutamate-gated chloride channel (GluCl) in complex with Fab and ivermectin*.
36. Kunishima N, Shimada Y, Tsuji Y, Sato T, Yamamoto M, Kumasaka T*, et al.* (2000): Structural basis of glutamate recognition by a dimeric metabotropic glutamate receptor. *Nature* 407: 971–977.
37. Geng Y, Xiong D, Mosyak L, Malito DL, Kniazeff J, Chen Y*, et al.* (2012): Structure and functional interaction of the extracellular domain of human GABA(B) receptor GBR2. *Nature neuroscience* 15: 970–978.

38. Wu H, Wang C, Gregory KJ, Han GW, Cho HP, Xia Y*, et al.* (2014): Structure of a class C GPCR metabotropic glutamate receptor 1 bound to an allosteric modulator. *Science (New York, N.Y.)* 344: 58–64.
